# Treatment of obstructive sleep apnea in high risk pregnancy: a multicenter randomized controlled trial

**DOI:** 10.1186/s12931-023-02445-y

**Published:** 2023-06-27

**Authors:** Visasiri Tantrakul, Atiporn Ingsathit, Somprasong Liamsombut, Sasivimol Rattanasiri, Prapun Kittivoravitkul, Nutthaphon Imsom-Somboon, Siwaporn Lertpongpiroon, Surasak Jantarasaengaram, Werapath Somchit, Worakot Suwansathit, Janejira Pengjam, Sukanya Siriyotha, Panyu Panburana, Christian Guilleminault, Aroonwan Preutthipan, John Attia, Ammarin Thakkinstian

**Affiliations:** 1 Department of Clinical Epidemiology and Biostatistics, Faculty of Medicine, Ramathibodi Hospital, Mahidol University, Bangkok, Thailand; 2 Division of Sleep Medicine, Department of Medicine, Faculty of Medicine, Ramathibodi Hospital, Mahidol University, Bangkok, Thailand; 3 Division of Pulmonary and Critical Care, Department of Medicine, Faculty of Medicine, Ramathibodi Hospital, Mahidol University, Bangkok, Thailand; 4 Ramathibodi Hospital Sleep Disorder Center, Mahidol University, Bangkok, Thailand; 5 Division of Pulmonary and Critical Care, Department of Medicine, Phramongkutklao Hospital, Bangkok, Thailand; 6 Division of Maternal Fetal Medicine, Department of Obstetrics and Gynecology, Phramongkutklao Hospital, Bangkok, Thailand; 7 Chest Unit, Rajavithi Hospital, Bangkok, Thailand; 8 Department of Obstetrics and Gynecology, Rajavithi Hospital, College of Medicine, Rangsit University, Bangkok, Thailand; 9 Division of Maternal Fetal Medicine, Department of Obstetrics and Gynecology, Faculty of Medicine, Ramathibodi Hospital, Mahidol University, Bangkok, Thailand; 10 Sleep Medicine Division, Stanford University, Redwood City, CA USA; 11 Division of Pediatric Pulmonary, Department of Pediatrics, Faculty of Medicine, Ramathibodi Hospital, Mahidol University, Bangkok, Thailand; 12 School of Medicine and Public Health, Faculty of Health and Medicine, The University of Newcastle, Callaghan, Australia

**Keywords:** Obstructive sleep apnea, Pregnancy, Continuous positive airway pressure (CPAP), Blood pressure, Preeclampsia

## Abstract

**Background:**

Obstructive sleep apnea (OSA) during pregnancy is a risk factor for preeclampsia possibly through a link to placental physiology. This study evaluates the efficacy of continuous positive airway pressure (CPAP) on the modulation of blood pressure and the reduction in preeclampsia in women with high-risk pregnancy and OSA.

**Methods:**

A multicenter open-label, randomized controlled trial comparing CPAP treatment versus usual antenatal care was conducted in three academic hospitals in Bangkok, Thailand. Participants included singleton pregnant women aged older than 18 years with any high-risk condition (i.e., chronic hypertension, obesity, history of preeclampsia or gestational diabetes in the previous pregnancy, or diabetes), and OSA (respiratory disturbance index 5–29.99 events/hour by polysomnography), who presented either in the first trimester (gestational age, GA 0–16 weeks) or subsequently developed OSA during the 2nd trimester (GA 24–28 weeks). The primary endpoint was blood pressure during antenatal care. Secondary endpoints included the incidence of preeclampsia. An intention-to-treat analysis was performed with additional per-protocol and counterfactual analyses for handling of nonadherence.

**Results:**

Of 340 participants, 96.5% were recruited during the first trimester. Thirty participants were later excluded leaving 153 and 157 participants in the CPAP and usual-care groups for the modified-intention-to-treat analysis. CPAP adherence rate was 32.7% with average use of 2.5 h/night. Overall, CPAP treatment significantly lowered diastolic blood pressure (DBP) by − 2.2 mmHg [95% CI (− 3.9, − 0.4), *p* = 0.014], representing approximately − 0.5 mmHg per hour of CPAP use [95%CI (− 0.89, − 0.10), *p* = 0.013]. CPAP treatment also altered the blood pressure trajectory by continuously lowering DBP throughout pregnancy with mean differences (95% CI) of − 3.09 (− 5.34, − 0.93), − 3.49 (− 5.67, − 1.31) and − 3.03 (− 5.20, − 0.85) mmHg at GA 18–20, 24–28, and 32–34 weeks, respectively compared to 0–16 weeks. Preeclampsia rate was 13.1% (20/153 participants) in the CPAP and 22.3% (35/157 participants) in the usual-care group with a risk difference (95% CI) of − 9% (− 18%, − 1%, *p*-value = 0.032) and a number-needed-to-treat (95% CI) of 11 (1, 21).

**Conclusions:**

CPAP treatment in women with even mild-to-moderate OSA and high-risk pregnancy demonstrated reductions in both DBP and the incidence of preeclampsia. CPAP treatment also demonstrated a sustained reduction in DBP throughout gestation.

*Trial registration* ClinicalTrial.GovNCT03356106, retrospectively registered November 29, 2017.

**Supplementary Information:**

The online version contains supplementary material available at 10.1186/s12931-023-02445-y.

## Background

Preeclampsia is a leading cause of maternal and fetal morbidity and mortality [1]. Obstructive sleep apnea (OSA) during pregnancy has been identified as a risk factor for preeclampsia, and gestational hypertension [2, 3]. Furthermore, with the overlapping risks for OSA and preeclampsia (i.e., obesity, chronic hypertension, or diabetes), OSA prevalence has been reported to be as high as 24–60% in cohorts including pregnant women with these risk factors [4–7], compared to 3.5–8.5% across trimesters in pregnancies generally [8]. During pregnancy, development [9] or exacerbation of preexisting-OSA can occur due to physiological and hormonal changes which lead to narrowing of the upper airway [10].

OSA is characterized by repetitive upper airway collapse during sleep leading to apneas/hypopneas, causing oxygen desaturation, arousals, sympathetic activation, and endothelial dysfunction [11, 12]; these pathophysiologic effects have been associated with the development of preeclampsia, possibly via abnormal placental physiology [13–15]. Furthermore, partial airway obstruction reflected in reports of snoring [9, 16, 17] and airflow limitation from polysomnography [17, 18] are highly prevalent during pregnancy [16–19]; in combination with apneas/hypopneas, this has been associated with adverse perinatal outcomes [2, 3, 16–18]. Thus the threshold to treat OSA during pregnancy may be below that for treatment of OSA in the general population [20, 21].

Although, continuous positive airway pressure (CPAP) is a standard treatment for OSA in general population [21], there is limited data regarding the efficacy and safety of CPAP treatment during pregnancy. Given that the expected benefits may outweigh the possible CPAP-related risks (including complex sleep apnea which has not been reported, or worsened sleep disruption), CPAP treatment has been used in pregnant women [22, 23].

CPAP treatment, with the elimination of airflow limitation in preeclamptic women, resulted in significant reduction of blood pressure (BP) [23]. Furthermore, increased airflow limitation during pregnancy was associated with subsequent preeclampsia [17]. Hemodynamic responses to obstructive respiratory events cause substantial transient increases in BP, both in normotensive and hypertensive pregnant women [19]. Therefore, we hypothesized that CPAP treatment in pregnant women with OSA may improve maternal hemodynamics leading to lower risks of gestational hypertensive disorders. Consistent with this, two recent non-randomized cohort studies showed that CPAP treatment could reduce the incidence of hypertensive complications in high-risk pregnant women with OSA [24, 25]. The efficacy of CPAP treatment in gestational OSA has been evaluated in only a few small randomized controlled trials (RCT), with inconclusive findings [26–28]. Our study aimed to evaluate the efficacy of CPAP treatment in high-risk pregnancy with mild-to-moderate OSA in reducing BP and hypertensive disorders in pregnancy, using a robust and well-powered randomized controlled trial design.

## Methods

### Study design and oversight

We conducted a multicenter, open-label, parallel-group RCT at three academic hospitals in Bangkok, Thailand. Methodologic details of the design and analysis plan have been registered via ClinicalTrial.gov (NCT03356106) and are provided in the full protocol in the supplement (the Additional file 1). Ramathibodi Hospital was responsible for the overall conduct and oversight of the study for all sites. The trial protocol was approved by all participating sites’ ethics committees listed in the supplement (the Additional file 2). All participants provided written informed consent.

### Patients and procedures

Pregnant women attending antenatal care at all collaborating hospitals were recruited for OSA screening if they met all of the following inclusion criteria: (1) singleton high-risk pregnant woman aged > 18 years without significant medical conditions (separate from those used as inclusion criteria), including immunocompromised status, chronic infection (HIV infection, or tuberculosis), chronic lung, cardiac or kidney disease, thyroid disease, or neuromuscular disease; (2) gestational age (GA) < 16 weeks for 1st-trimester, or GA 24–28 weeks for 2nd trimester; and (3) Thai nationality, proficient in Thai language. High-risk pregnancy was defined as any of the following: (a) chronic hypertension defined by preexisting-hypertension or hypertension diagnosed before 20 weeks’ gestation; (b) history of preeclampsia or gestational hypertension in the previous pregnancy; (c) obesity defined by pre-pregnancy body mass index (BMI) ≥ 27.5 kg/m^2^ as per Asian criteria [29]; (d) history of gestational diabetes (GDM) in the previous pregnancy or; (e) diabetes. All potential eligible participants were scheduled and screened for OSA by performing full-night, type-2 in-laboratory polysomnography (SomnoTouchResp®) during 1st-trimester. Subsequent 2nd-trimester-polysomnography was repeated to detect new-onset OSA if 1st-trimester-polysomnography showed respiratory disturbance index (RDI) < 5 events/hour. Participants were classified as OSA if they had respiratory RDI 5–29.99 events/hour either detected initially during the first-trimester (GA 0–16 weeks) or the 2nd-trimester (GA 24–28 weeks). Initially, presence of snoring (frequent snoring ≥ 3 times/week) was listed as one of the inclusion criteria, but fewer participants reported snoring, thus OSA criteria were entirely based on objective RDI criteria.

Sleep stages and respiratory scorings were performed according to the American Academy of Sleep Medicine (AASM) 2012 guidelines [30], see protocol (Additional file 1). Respiratory events were scored based on the AASM recommended criteria with each event duration being at least 10 s. Apnea was defined as a reduction of airflow signal at least 90% from baseline; hypopnea was defined as a reduction in nasal pressure transducer signal at least 30% from baseline that was associated with either O_2_ desaturation ≥ 3% or arousal (AASM-recommended hypopnea). Additionally, respiratory effort related arousal (RERA) was defined as a sequence of breaths characterized by inspiratory airflow flattening of the nasal pressure leading to arousal.

RDI was defined as a number of apneas, hypopneas, and RERA per hour of sleep; apnea–hypopnea index (AHI) was calculated as a number of apneas and hypopneas per hour of sleep [30]. Scoring was done by two independent scorers. Discrepancies between scorers were adjudicated by a 3rd scorer.

Exclusion criteria included: (1) severe OSA (RDI ≥ 30 events/hour) or significant oxygen desaturation < 80% during sleep; or (2) known OSA currently on CPAP treatment. For ethical reasons, CPAP treatment was offered to severe cases due to potential harms to the mother and fetus and the possible benefit of treatment.

### Randomization and interventions

Randomization was stratified by trimesters with varying block sizes of 4–8. After each participant agreed to participate and signed informed consent, clinical data were entered into a centralized computer system for automatic randomization sequence generation and subsequent immediate allocation. Participants receiving CPAP initiated treatment nightly until delivery. An auto-adjusted CPAP (auto-CPAP: Phillips-Respironics REMstarAutoM®/Dreamstation®) with heated-humidification delivered via nasal mask was used throughout pregnancy with pressure range between 4 and 15 cmH_2_O to overcome the continuous change of pregnancy. Data (i.e., hour-use, AHI, leakage) recorded within the memory-card were downloaded and discussed with participants during each scheduled antenatal visit. Weekly telephone contact was made with each participant for troubleshooting and encouraging adherence by sleep technologists. Average-CPAP use throughout the study in hours/night was calculated for each participant by dividing the cumulative use time by the numbers of days from randomization until delivery. An average-CPAP use ≥ 4 h/night was considered good adherence and < 4 h/night was considered non-adherence. This categorization was used in the post-hoc analyses addressing non-adherence. Both CPAP- and usual-care groups received similar antenatal care, follow-up plans and advice on sleep hygiene during pregnancy.

### Study measurements

Data were collected during GA 18–20, 24–28, 32–34 weeks during regular scheduled antenatal care and delivery using case record forms that captured demographic data, sleep questionnaires, primary and secondary endpoints, and CPAP adherence. BP measurements by sphygmomanometer were obtained twice on both arms in resting-sitting positions at least 15 min apart; these were then averaged for the analyses. Treating obstetricians were not involved in the conduct of the research. Pregnancy complications were diagnosed by treating obstetricians according to the Report of the American College of Obstetricians and Gynecologists’ Task Force on hypertension in pregnancy [31, 32], see protocol. A case record form with checklist criteria was used for retrieval of pregnancy outcomes to ensure accurate diagnoses.

### Study endpoints

The primary outcomes were systolic (SBP) and diastolic BP (DBP) measured during the scheduled antenatal care visit (between 10 am–12 pm) during each specific gestational time-point. For participants randomized during the 2nd-trimester, only outcome data after randomization was included for analyses. Secondary outcomes included the incidence of hypertensive disorders in pregnancy consisting of preeclampsia, and gestational hypertension [31, 32]. Other maternal and fetal outcomes, including preterm birth, fetal growth restriction, and emergency cesarean-section, were also analyzed both as individual and composite outcomes.

GDM was not included in the endpoint analyses as initially planned because it was one of the inclusion criteria, and most cases were detected early before randomization as routine practice in participating sites given the high GDM prevalence in Asians [33]. However, participants with chronic hypertension were still included for the analysis of preeclampsia based on any findings of new developments of proteinuria, thrombocytopenia, liver dysfunction, renal insufficiency, or symptoms suggestive of preeclampsia [31, 32].

### Statistical analysis

Sample size was calculated based on a 1:1 ratio of CPAP- and usual-care groups, assuming the BP lowering-effect of CPAP in the general population with OSA was 2.5 mmHg (from meta-analysis data) [34]. Using values from a previous study in pregnancy with OSA demonstrating DBP of 92.7 (standard deviation, SD 7.4) [35], type I and II errors of 5 and 20%, and assuming loss to follow-up of 20%, a total of 334 participants were required (167 each arm). This sample size gave 80% power to detect a 12–15% reduction of the incidence of hypertensive disorders in pregnancy.

Data were described using mean and SD or median and interquartile range (IQR) as appropriate for continuous variables, and percentage for categorical variables. Baseline characteristics were compared between treatment groups using t-test and χ^2^-test (or Fisher-Exact where appropriate). The statistical analysis for the primary objective was performed based on a modified-intention-to-treat analysis. Two additional post-hoc approaches (per-protocol, and counterfactual analyses) were also performed. A linear mixed-effect model, taking into account the repeated measurements at each gestational timepoint, was used to analyze the primary outcome after randomization considering the continuous changes of BP throughout pregnancy. The counterfactual approach was performed using instrumental variable analysis to assess what potential outcomes would have been if participants had complied with the assigned intervention, known as a complier-average treatment effect [36]. A two-stage least squares approach with bivariate probit was applied, considering the assigned intervention and that actually received as the instrumental and endogenous variables, respectively. Statistical analyses were performed using STATA version 16.1 (StataCorp®, TX), with a significance threshold *p*-value < 0.05 (2-sided).

## Results

### Participants’ baseline characteristics

**Fig. 1:**
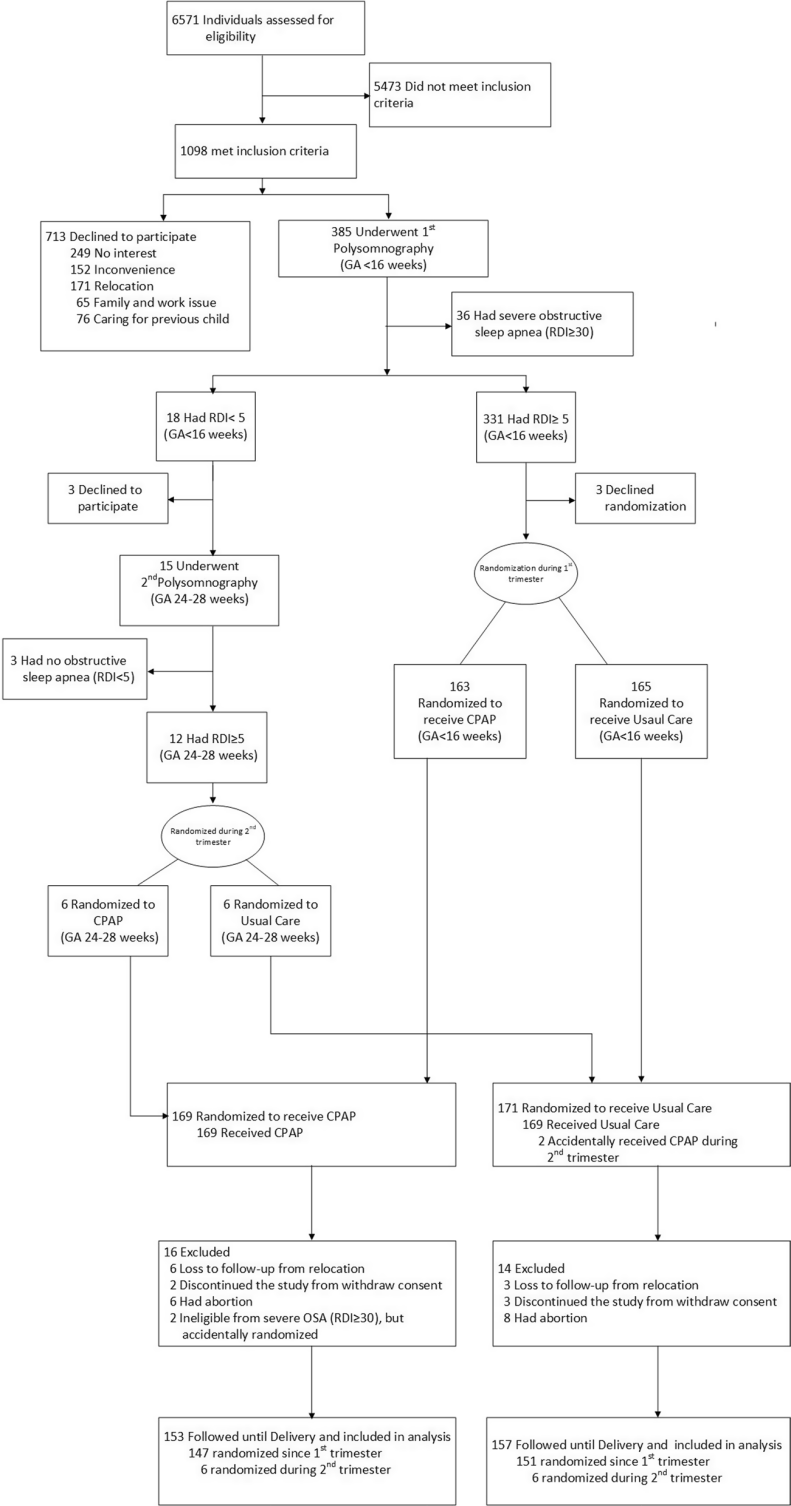
Consort flow diagram for screening, randomization, and follow-up analyses. *CPAP* continuous positive airway pressure; *GA* gestational age; *OSA* obstructive sleep apnea; *RDI* respiratory disturbance index (events/hour). All participants were initially enrolled only in the 1st trimester. Diagnosis of obstructive sleep apnea involved full-night, type-2 polysomnography (SomnoTouchResp®) during 1st trimester (GA < 16 weeks). Second polysomnography during 2nd trimester (GA 24–28 weeks) was repeated in participants if their 1st trimester RDI was < 5 events/hour. Participant with RDI between 5 and 29.99 events/hour at either testing were randomized. Only participants that had delivery outcomes were included in the modified intention-to-treat analyses

Of the 6571 pregnant women screened, only 1098 were potentially eligible, but 713 declined to participate (reasons described in Fig. 1) leaving 385 patients who agreed to participate and who underwent overnight-polysomnography between November 2016 and June 2019. During the 1st-trimester-polysomnography, 331 participants had RDI 5–29.99 events/hour and thus were randomized, while only18 participants had RDI < 5 and were candidates for the 2nd-trimester-polysomnography. Of these, 3 participants declined to further participate leaving 15 participants for subsequent 2nd-trimester-polysomnography, of which 12 had RDI 5–29.99 events/hour and were randomized. Of all participants, 154 (49.7%) reported frequent snoring, thus eligibility was 100% based on RDI ≥ 5 events/hour. In summary, 169 and 171 participants were randomly assigned to receive CPAP plus usual-care, and usual-care alone, respectively. A total of 16 and 14 participants in the CPAP-and-usual-care groups respectively discontinued the study, leaving 153 and 157 in CPAP-and-usual-care groups for a modified-intention-to-treat analysis. Only participants that had delivery outcomes were included in the modified intention-to-treat analyses. Reasons for exclusion were abortions (n = 14) occurring before 20 weeks gestations and within a week after randomization; withdrawal of consent immediately after randomization (n = 5); loss to follow-up from relocation (n = 9); and ineligibility from severe OSA accidentally randomized from administrative error (n = 2). The study was completed with the last delivery in November 2019; median time (IQR) in study was 171 (144, 189) days.

Baseline characteristics of participants are described in Table 1; participants had mean age of 32.8 years and GA at randomization was 14.8 weeks, 105 (33.9%) were nulliparous and 210 (67.7%) were obese. Mean RDI and AHI were 14.5, and 7.1 events/hour, respectively, and average oxygen saturation and desaturation nadir during sleep were 96.7% and 89.3%. Baseline demographic data, medications, and polysomnographic data were balanced between both groups (Tables 1, 2, 3). Depending on the treating obstetricians, anti-hypertensive medications (mainly methyl-dopa and/or hydralazine) were prescribed to 24 participants (12 each group) in similar initial/final dosages with 8 participants (4 each group) requiring adjustments after 24–26 weeks gestation (Table 3). Antihypertensive drug use between the two groups was not significantly different (7.8% vs 7.6%), but the usual-care group required a combination of methyl-dopa/hydralazine [8 (66.7%) vs 2 (16.7%), *p*-value = 0.013], additional 3rd anti-hypertensive medication [3 (25%) vs 1 (8.3%), p = 0.273], and MgSO_4_ for stabilization during delivery [10 (83.33%) vs 4 (33.33%), p = 0.013] more than the CPAP-group. Missing data ranged from 0 to 37.4% (hemoglobin A1C) (Additional file 2: Table S1). However, data for the primary and secondary outcomes (i.e., BPs, hypertensive disorders in pregnancy) were not missing, therefore data imputation was not performed.

**Table 1:** Baseline characteristics of the participants by intervention groups

Characteristics	CPAP group N = 153	Usual-care group N = 157	*p *value
Age (year), mean (SD)	32.9 ± 5.3	32.6 ± 5.1	0.553
Gestational age at enrollment (week), mean (SD)	11.18 ± 4.0	10.26 ± 3.14	0.908
Gestational age at randomization (week), mean (SD)	15.2 ± 4.8	14.5 ± 4.5	0.180
Nulliparous, no. (%)	49 (32.0)	56 (35.7)	0.498
Anthropometric measurement			
Pre-pregnancy body mass index (kg/m^2^)^a^, mean (SD)	29.2 ± 5.8	29.6 ± 5.5	0.546
Body mass index at enrollment (kg/m^2^), mean (SD)	30.1 ± 5.9	30.4 ± 5.5	0.722
Neck circumference (cm), mean (SD)	35.4 ± 3.3	35.4 ± 3.2	0.977
Waist circumference (cm), mean (SD)	97.7 ± 12.6	97.7 ± 11.0	0.971
Type of inclusion criteria^b^, no. (%)			
Obesity^c^	99 (64.7)	111 (70.7)	0.259
Chronic hypertension	27 (17.7)	23 (14.7)	0.473
Pre-existing hypertension before pregnancy	25 (16.3)	21 (13.4)	0.463
Hypertension occurring before 20 weeks of gestation	10 (6.5)	8 (5.1)	0.588
History of preeclampsia in previous pregnancy	13 (8.5)	14 (8.9)	0.896
History of gestational hypertension in previous pregnancy	3 (2.0)	4 (2.5)	0.728
Known case of diabetes mellitus	12 (7.8)	13 (8.3)	0.888
History of gestational diabetes in previous pregnancy	29 (19.0)	28 (17.8)	0.799
Gestational diabetes detected early	72 (47.1)	70 (44.6)	0.662
Epworth Sleepiness Scale^d^, median (IQR)	8.5 (5.0, 11.0)	8.0 (5.0, 12.0)	0.093
Epworth Sleepiness Scale ≥ 11, no., %	48 (31.4)	51 (32.5)	0.833
Report of frequent snoring ≥ 3 times/week^e^, no. (%)	76 (49.7)	78 (49.7)	0.999
Systolic blood pressure (mmHg), mean (SD)	116.9 ± 1.3	116.0 ± 1.1	0.574
Diastolic blood pressure (mmHg), mean (SD)	73.5 ± 0.9	73.9 ± 0.7	0.706
Mean blood pressure (mmHg), mean (SD)	88.1 ± 0.8	88.1 ± 1.0	0.989
Fasting plasma glucose (mg/dL), mean (SD)	99.3 ± 28.1	101.4 ± 35.8	0.581
Hemoglobin A1C (mg/dL), mean (SD)	5.4 ± 0.8	5.6 ± 1.0	0.211

*CPAP* continuous positive airway pressure; *IQR* interquartile range; *SD* standard deviation

^a^Body-mass index is the weight in kilograms divided by the square of the height in meters (kg/m^2^)

^b^Medical history was self-reported and determined through a review of medical records

^c^Obesity is defined by pre-pregnancy body-mass index that is equal to or greater than 27.5 kg/m^2^ for Asian cut-off threshold

^d^The Epworth Sleepiness Scale ranges from 0 to 24, with higher scores indicating greater sleepiness; a score higher than 10 indicates pathologic sleepiness

^e^Snoring was reported by the participants on a questionnaire

**Table 2:** Polysomnographic characteristics of participants by intervention groups

Polysomnographic parameter	CPAP group N = 153	Usual-care group N = 157	*p *value
Total sleep time (hour), mean (SD)	6.0 ± 1.0	6.2 ± 0.8	0.044
Sleep efficiency (%), mean (SD)	84.7 ± 11.6	85.9 ± 8.8	0.329
Sleep latency (minute), median (IQR)	14.4 (7.8, 25.7)	13.6 (7.5, 27.8)	0.730
Stage N1 and N2 (%), mean (SD)	62.6 ± 8.3	63.4 ± 8.3	0.371
Stage N3 (%), mean (SD)	20.6 ± 7.7	19.2 ± 7.3	0.085
Stage REM (%), mean (SD)	16.8 ± 5.0	17.4 ± 5.2	0.276
Sleep time during supine position (%), mean (SD)	69.1 ± 24.8	65.6 ± 24.2	0.080
Respiratory disturbance index (RDI)^a^ (events/hour), mean (SD)	14.1 ± 6.6	14.9 ± 6.6	0.948
Apnea–hypopnea index (AHI)^b^ (events/hour), median (IQR)^b^	7.0 (4.4, 12.5)	7.2 (4.4, 13.5)	0.813
Apnea index (events/hour), median (IQR)	0.2 (0.0, 1.2)	0.3 (0.0, 0.9)	0.262
Hypopnea index (events/hour), median (IQR)	6.4 (4.2, 10.1)	6.9 (4.0, 11.8)	0.453
Apnea–hypopnea index in REM sleep (events/hour), median (IQR)	16.1 (7.6, 31.3)	14.1 (7.6, 24.0)	0.249
Apnea–hypopnea index in NREM sleep (events/hour), median (IQR)	5.1 (2.6, 11.4)	6.8 (2.8, 11.8)	0.041
Apnea–hypopnea index during supine position (events/hour), median (IQR)	13.9 (9.5, 20.9)	15.6 (8.7, 21.2)	0.176
Apnea–hypopnea index during non-supine position (events/hour), median (IQR)	5.1 (2.6, 8.5)	5.1 (2.0, 10.2)	1.000
Oxygen desaturation index^c^ (events/hour), median (IQR)	1.9 (0.6, 4.3)	2.6 (0.9, 5.2)	0.372
Average oxygen saturation (%), mean (SD)	96.7 ± 1.1	96.6 ± 1.0	0.455
Minimum oxygen saturation (%), mean (SD)	89.4 ± 4.2	89.2 ± 3.9	0.686
Time oxygen saturation under 90% (minutes), median (IQR)	0 (0, 0.06)	0 (0, 0.07)	0.359
Obstructive sleep apnea characteristics			
Severity^d^, no. (%)			
Mild	71(46.4)	73(46.5)	0.987
Moderate	82(53.6)	84(53.5)	
In-laboratory polysomnography, no. (%)	152 (99.4)	156 (99.4)	1.0
Agreement between scorers (%), mean (SD)	89.0 ± 10.2	90.0 ± 11.8	0.433

*CPAP* continuous positive airway pressure; *IQR* interquartile range; *SD* standard deviation

^a^The respiratory disturbance index (RDI) is the number of apnea, hypopnea and respiratory-event related arousal events per hour of sleep

^b^The apnea–hypopnea index is the number of apnea, and hypopnea events per hour of sleep

^c^The oxygen desaturation index is the number of times per hour of sleep during the oximeter recording that the oxygen saturation drops by at least 3 percentage points from baseline

^d^Severity of obstructive sleep apnea is classified as mild (respiratory disturbance index, RDI ≥ 5 and < 15 events/hour) and moderate (RDI ≥ 15 and < 30 events/hour)

**Table 3:** Medication use by intervention groups during pregnancy

Medication use, no. (%)	CPAP group N = 153	Usual-care group N = 157	*p *value
Antihypertensive agent use, no. (%)	12 (7.8)	12 (7.6)	0.947
Methyldopa, no. (%)	11 (7.2)	12 (7.6)	0.307
Initial dose (mg/day), median (IQR)	500 (250, 750)	500 (500, 750)	0.129
Final dose (mg/day), median (IQR)	750 (500, 750)	500 (250, 750)	0.339
Hydralazine, no. (%)	3 (25.0)	8 (66.7)	0.041
Initial dose (mg/day), median (IQR)	62.5 (37.5, 87.5)	50.0 (37.5, 75)	0.441
Final dose (mg/day), median (IQR)	75 (37.5, 200)	75 (50, 75)	0.267
Combined methyldopa and hydralazine, no. (%)	2 (16.7)	8 (66.7)	0.013
Additional 3rd anti-hypertensive medication, no. (%)	1 (8.3)	3 (25.0)	0.273
MgSO_4_ stabilization during delivery, no. (%)	4 (33.3)	10 (83.3)	0.013
Insulin—no. (%)	33 (21.6)	32 (20.4)	0.798
Insulin dose (u/day), median (IQR)	32 (3, 160)	46 (6, 140)	0.217
Other medications			
Aspirin, no. (%)	35 (22.9)	40 (25.5)	0.593
Calcium supplement, no. (%)	53 (34.6)	50 (31.9)	0.602
Vitamin D supplement, no. (%)	6 (4.8)	2 (1.7)	0.142

*CPAP* continuous positive airway pressure; *IQR* interquartile range

### Adherence to intervention

Overall, the intervention group had mean average-CPAP use of 2.5 (SD 2.5) and median of 1.7 (IQR 0.2, 4.5) hours/night; only 50 (32.7%) participants were adherent to treatment (defined as average-CPAP use ≥ 4 h/night). The minimum, maximum, and 90th percentile pressures of auto-CPAP were 4.9 (1.3), 8.2 (1.8), and 6.3 (1.3) cmH_2_O, respectively. Clinical characteristics between CPAP adherent- and non-adherent participants were not different except for GA at randomization (13.6 ± 3.6 vs 15.9 ± 5.1 weeks, *p*-value = 0.004), see Additional file 2: Tables S2, S3.

### Effect of CPAP on BPs

Using the modified-intention-to-treat approach, the overall marginal mean SBP/DBP and mean arterial pressure (MAP) were estimated by intervention group within each specific GA stratum. Mean DBP and MAP were significantly lower in the CPAP than usual-care groups with mean differences (95% CI) of − 2.2 (− 3.9, − 0.4), and − 2.1 (− 3.9, − 0.2) mmHg, respectively. After adjusting for underlying hypertension status, anti-hypertensive medication use and gestational age, the results remained significant, see Table 4.

**Table 4:** Primary outcomes on blood pressures using the modified intention-to-treat analysis

Modified intention-to-treat analysis												
Mean (SE)^a^	CPAP (n = 153)			Usual-care (n = 157)			Overall mean		Intergroup difference (95%CI)^c^	*p *value	Adjusted intergroup difference (95%CI)^d^	*p *value
	18–20 weeks^b^	24–28 weeks	32–34 weeks	18–20 weeks^b^	24–28 weeks	32–34 weeks	CPAP vs control					
SBP	114.7 (1.0)	112.5 (1.0)	115.2 (1.0)	116.7 (1.0)	114.4 (1.0)	117.2 (1.0)	114.1 (1.0)	116.1 (0.9)	− 1.9 (− 4.5, 0.6)	0.148	− 2.08 (− 4.41, 0.26)	0.082
DBP	70.4 (0.7)	69.3 (0.7)	71.3 (0.7)	72.6 (0.7)	71.5 (0.7)	73.8 (0.7)	70.4 (0.6)	72.5 (0.6)	− 2.2 (− 3.9, − 0.4)	0.014	− 2.22 (− 3.70, − 0.75)	0.003
MAP	85.2 (0.8)	83.7 (0.8)	86.0 (0.8)	87.3 (0.7)	85.8 (0.7)	88.0 (0.7)	85.0 (0.7)	87.0 (0.7)	− 2.1 (− 3.9, − 0.2)	0.034	− 2.17 (− 3.83, − 0.52)	0.010

*CPAP* continuous positive airway pressure; *DBP* diastolic blood pressure; *MAP* mean arterial pressure; *SBP* systolic blood pressure; *SE* standard error; *95%CI* 95% confidence interval

^a^Primary outcome on systolic and diastolic blood pressures are shown as means and SE estimated at each time point from the mixed-effect linear regression

^b^Blood pressure data during 18–20 weeks gestation from participants who were randomized during 2nd trimester in both CPAP (n = 6) and usual-care groups (n = 6) were not included in the analyses

^c^Intergroup difference is calculated as difference of marginal means (overall mean) between CPAP compared to the usual-care groups using mixed-effect linear regression model after randomization presented as mean difference and 95% CI

^d^Adjusted intergroup difference is calculated as difference of marginal mean between CPAP compared to the usual-care groups using a mixed-effect linear regression model after randomization presented as mean difference and 95%CI adjusted with underlying chronic hypertension status, anti-hypertensive medication use and gestational age

**Fig. 2:**
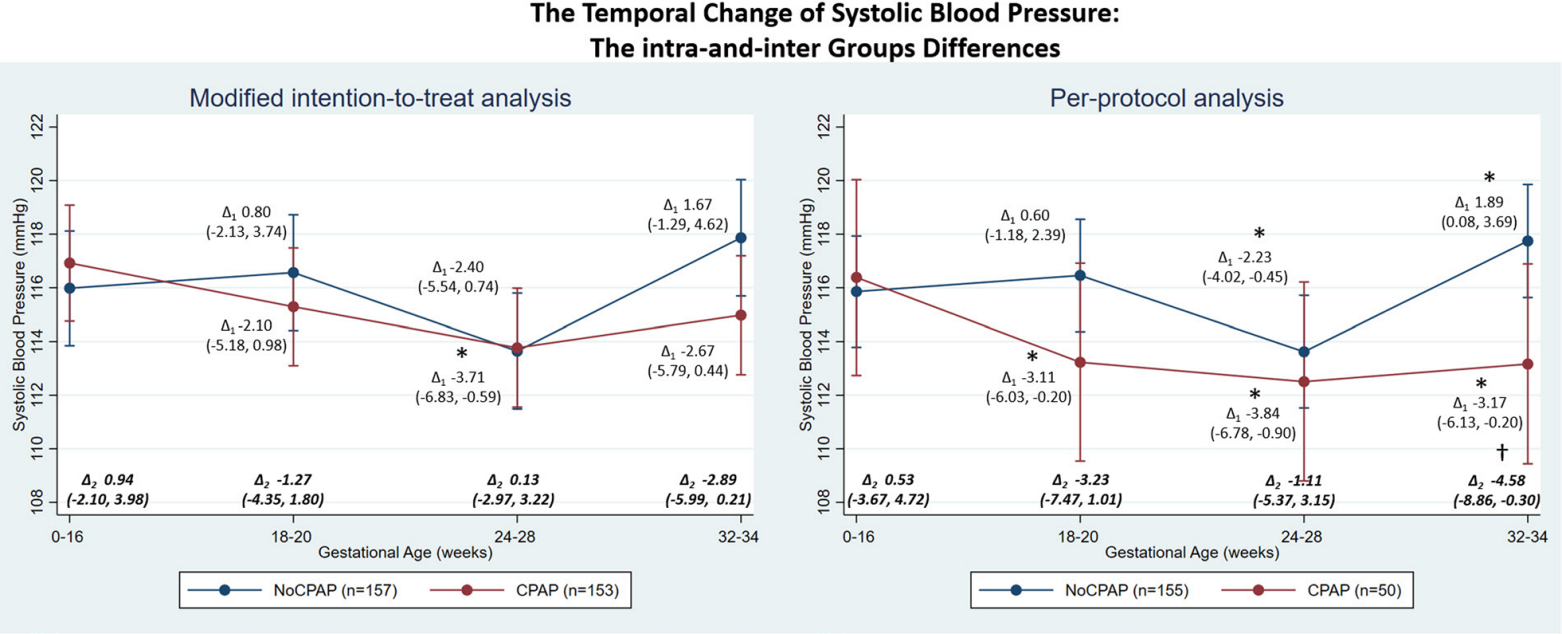
Temporal changes of systolic blood pressure during pregnancy in CPAP and usual-care (no CPAP) groups during 18–20, 24–28, and 32–34 weeks gestation compared to baseline (gestational age < 16 weeks) in the modified intention-to-treat and per-protocol analyses. *Note* The BP nadir-point occurred at 24–28 weeks in both CPAP and usual-care groups. *Modified intention-to-treat analysis* Intra-group changes: ∆_1_ = difference within groups compared to baseline; *Denotes p-value < 0.05**—**CPAP group (lower panel): 18–20 weeks vs baseline, p-value = 0.181; 24–28 weeks vs baseline, p-value = 0.020; 32–34 weeks vs baseline, p-value = 0.093. Usual-care group (upper panel): 18–20 weeks vs baseline, p-value = 0.592; 24–28 weeks vs baseline, p-value = 0.108; 32–34 weeks vs baseline, p-value = 0.268. Inter-group changes: ∆_2_ = difference between groups during each timepoints; ^†^Denotes *p*-value < 0.05—CPAP vs usual-care groups: baseline, p-value = 0.545; 18–20 weeks, p-value = 0.417; 24–28 weeks, p-value = 0.936; 32–34 weeks, p-value = 0.068. *Per-protocol analysis* Intra-group changes: ∆_1_ difference within groups compared to baseline; *Denotes *p*-value < 0.05—CPAP group (lower panel): 18–20 weeks vs baseline, p-value = 0.037; 24–28 weeks vs baseline, p-value = 0.010; 32–34 weeks vs baseline, p-value = 0.036. Usual-care group (upper panel): 18–20 weeks vs baseline, p-value = 0.507; 24–28 weeks vs baseline, p-value = 0.014; 32–34 weeks vs baseline, p-value = 0.040. Inter-group changes: ∆_2_ difference between groups during each timepoints; ^†^Denotes *p*-value < 0.05—CPAP vs usual-care groups: baseline, p-value = 0.805; 18–20 weeks, p-value = 0.135; 24–28 weeks, p-value = 0.609; 32–34 weeks, p-value = 0.036

**Fig. 3:**
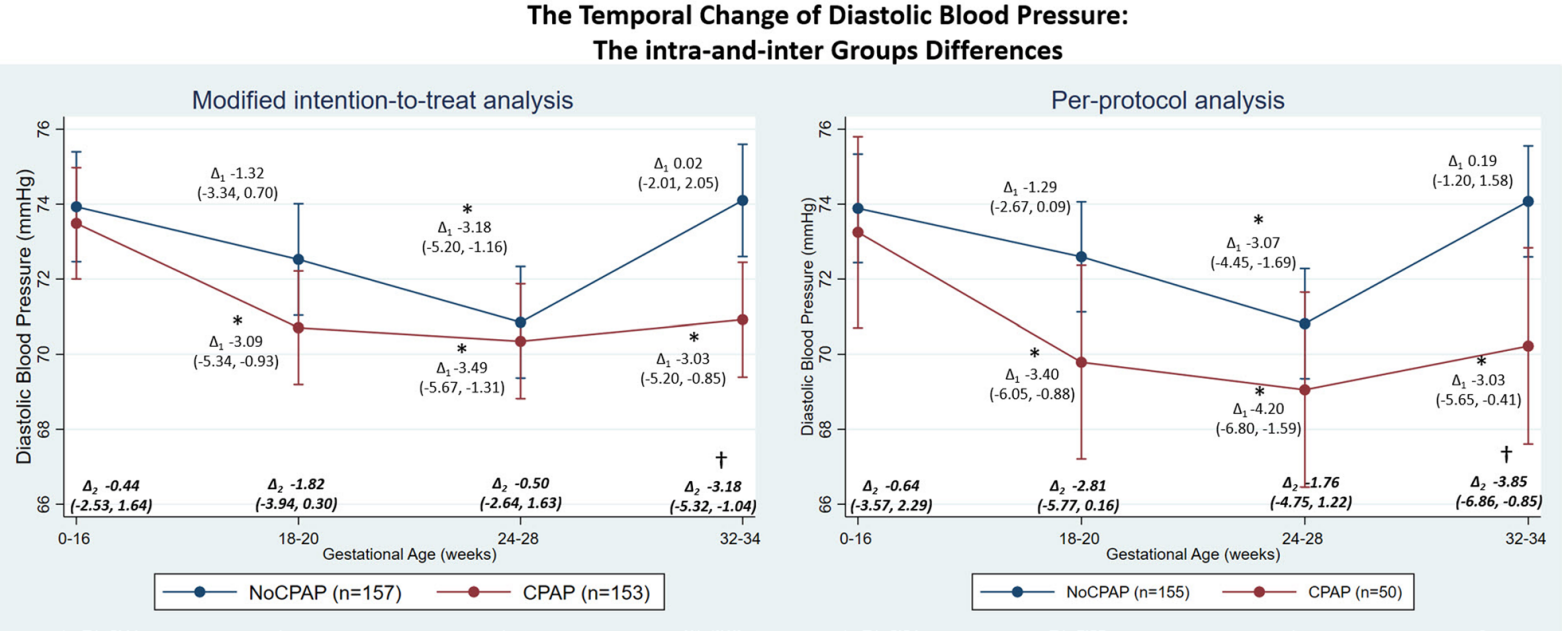
Temporal changes of diastolic blood pressure during pregnancy in CPAP and usual-care (no CPAP) groups during 18–20, 24–28, and 32–34 weeks gestation compared to baseline (gestational age < 16 weeks) in the modified intention-to-treat and per-protocol analyses. *Note* The BP nadir-point occurred at 24–28 weeks in both CPAP and usual-care groups. *Modified intention-to-treat analysis* Intra-group changes: ∆_1_ difference within groups compared to baseline; *Denotes *p*-value < 0.05—CPAP group (lower panel): 18–20 weeks vs baseline, p-value = 0.005; 24–28 weeks vs baseline, p-value = 0.002; 32–34 weeks vs baseline, p-value = 0.006. Usual-care group (upper panel): 18–20 weeks vs baseline, p-value = 0.199; 24–28 weeks vs baseline, p-value = 0.002; 32–34 weeks vs baseline, p-value = 0.987. Inter-group changes: ∆_2_ difference between groups during each timepoints; ^†^Denotes *p*-value < 0.05—CPAP vs usual-care groups: baseline, p-value = 0.677; 18–20 weeks, p-value = 0.092; 24–28 weeks, p-value = 0.643; 32–34 weeks, p-value = 0.004. *Per-protocol analysis* Intra-group changes: ∆_1_ difference within groups compared to baseline; *Denotes *p*-value < 0.05—CPAP group (lower panel): 18–20 weeks vs baseline, p-value = 0.009; 24–28 weeks vs baseline, p-value = 0.002; 32–34 weeks vs baseline, p-value = 0.023. Usual-care group (upper panel): 18–20 weeks vs baseline, p-value = 0.066; 24–28 weeks vs baseline, p-value < 0.001; 32–34 weeks vs baseline, p-value = 0.793. Inter-group changes: ∆_2_ difference between groups during each timepoints; ^†^Denotes *p*-value < 0.05—CPAP vs usual-care groups: baseline, p-value = 0.670; 18–20 weeks, p-value = 0.064; 24–28 weeks, p-value = 0.247; 32–34 weeks, p-value = 0.012

**Fig. 4:**
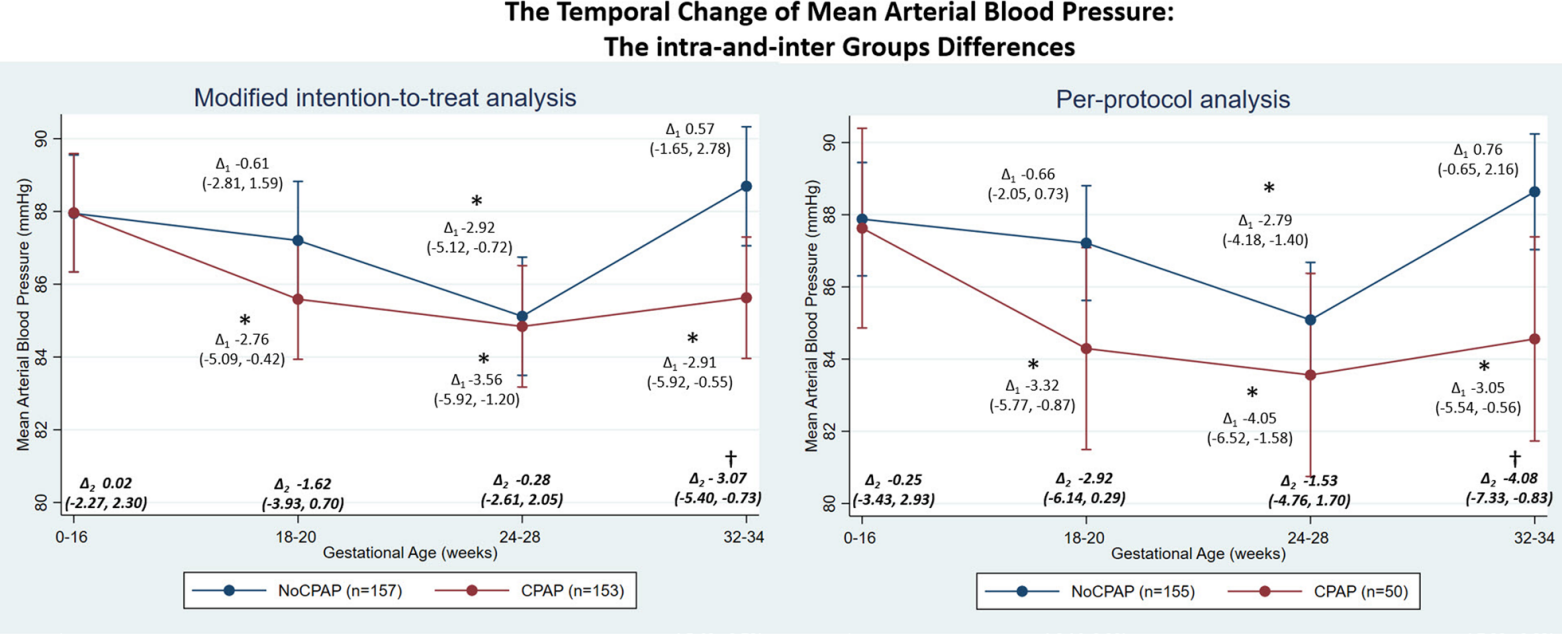
Temporal changes of mean blood pressure during pregnancy in CPAP and usual-care (no CPAP) groups during 18–20, 24–28, and 32–34 weeks gestation compared to baseline (gestational age < 16 weeks) in the modified intention-to-treat and per-protocol analyses. *Note* The BP nadir-point occurred at 24–28 weeks in both CPAP and usual-care groups. *Modified intention-to-treat analysis* Intra-group changes: ∆_1_ difference within groups compared to baseline; *Denotes *p*-value < 0.05—CPAP group (lower panel): 18–20 weeks vs baseline, p-value = 0.021; 24–28 weeks vs baseline, p-value = 0.003; 32–34 weeks vs baseline, p-value = 0.016. Usual-care group (upper panel): 18–20 weeks vs baseline, p-value = 0.585; 24–28 weeks vs baseline, p-value = 0.009; 32–34 weeks vs baseline, p-value = 0.616. Inter-group changes: ∆_2_ difference between groups during each timepoints; ^†^Denotes *p*-value < 0.05—CPAP vs usual-care groups: baseline, p-value = 0.988; 18–20 weeks, p-value = 0.172; 24–28 weeks, p-value = 0.815; 32–34 weeks, p-value = 0.010. *Per-protocol analysis* Intra-group changes: ∆_1_ difference within groups compared to baseline; *Denotes *p*-value < 0.05—CPAP group (lower panel): 18–20 weeks vs baseline, p-value = 0.008; 24–28 weeks vs baseline, p-value = 0.001; 32–34 weeks vs baseline, p-value = 0.016. Usual-care group (upper panel): 18–20 weeks vs baseline, p-value = 0.351; 24–28 weeks vs baseline, p-value < 0.001; 32–34 weeks vs baseline, p-value = 0.290. Inter-group changes: ∆_2_ difference between groups during each timepoints; ^†^Denotes *p*-value < 0.05—CPAP vs usual-care groups: baseline, p-value = 0.878; 18–20 weeks, p-value = 0.075; 24–28 weeks, p-value = 0.354; 32–34 weeks, p-value = 0.014

The temporal change in BP showed a mid-pregnancy fall with a nadir at GA 24–28 weeks, see intra-group difference in Figs. 2, 3, and 4. For the modified-intention-to-treat analysis, the SBP nadir was significant only in the CPAP, but not in the usual-care group (Fig. 2). DBP was lower throughout pregnancy in the CPAP-group with mean differences (95% CI) of − 3.09 (− 5.34, − 0.93), − 3.49 (− 5.67, − 1.31) and − 3.03 (− 5.20, − 0.85) mmHg at GA 18–20, 24–28, and 32–34 weeks, respectively when compared to GA < 16 weeks; a DBP reduction was only significant in the usual-care group at the GA 24–28 weeks nadir (Fig. 3). MAP was also lower in the CPAP-group at all time-points with the corresponding mean of − 2.76 (− 5.09, − 0.42), − 3.56 (− 5.92, − 1.20), and − 2.91 (− 5.92, − 0.55) mmHg; again, only the GA 24–28 weeks nadir was significant in the usual-care group (Fig. 4).

Considering the intergroup difference at the different time-points, CPAP treatment significantly lowered DBP [− 3.18 (− 5.32, − 1.04)] and MAP [− 3.07 (− 5.40, − 0.73)] mmHg at GA 32–34 week, resulting from the marked increase in BP after the nadir-point of 24–28 week in the usual-care group in contrast to the persistently lowered BP of the CPAP-group (Figs. 2, 3, 4). These trends were still consistent in subgroups of participants who did and did not take anti-hypertensive medications (Additional file 2: Figs. S1, S2, S3, S4). Furthermore, in those taking anti-hypertensive medication, the BP nadir-point and the increase thereafter occurred earlier at GA 18–20 weeks in the usual-care group. However, this early increase in BP was prevented by CPAP use (Additional file 2: Figs. S1, S2, S3, S4).

Every hour of CPAP use decreased SBP, DBP, and MAP by − 0.60 (− 1.19, − 0.01), − 0.50 (− 0.89, − 0.10), and − 0.53 (− 0.97, − 0.09) mmHg, respectively.

### Effect of CPAP on pregnancy outcomes

For the modified-intention-to-treat analysis, the incidence of hypertensive disorders in pregnancy was significantly lower in the CPAP than the usual-care group, with an incidence of 13.7% (21/153) and 24.8% (39/157), respectively (p = 0.012); the risk difference (95% CI) was − 11% (− 20%, − 2%), and number-needed-to-treat (NNT) (95%CI) was 9 (2, 16) (Table 5). The significance of this endpoint was driven largely by the incidence of preeclampsia, which was significantly lower in the CPAP compared to the usual-care group [13.1% (20/153) vs 22.3% (35/157), *p*-value = 0.032] with a risk difference (95% CI) of − 9% (− 18%, − 1%) and NNT (95% CI) of 11 (1, 21). In particular, CPAP significantly reduced preeclampsia at the late-onset (GA ≥ 34 weeks) but not early-onset (GA < 34 weeks) time-points (Table 5).

**Table 5:** Secondary outcomes on preeclampsia, pregnancy-induced hypertension using the modified intention-to-treat analysis

Endpoints	CPAP group	Usual-care group	Risk difference, % (95%CI)^a^	Number needed to treat (95%CI)^a^	*p *value
*Modified intention to treat analysis*	(*n* = *153*)	(*n* = *157*)			
Preeclampsia, no. (%)	20 (13.1)	35 (22.3)	− 9 (− 18, − 1)	11 (1, 21)	0.032
Severe preeclampsia^b^	13 (8.5)	22 (14.0)	− 6 (− 14, 13)	–	0.122
Early-onset preeclampsia^c^	4 (2.6)	4 (2.6)	0.1 (− 4, 3.5)	–	0.97
Late-onset preeclampsia^d^	16 (10.5)	31 (19.8)	− 9 (− 17, − 1.4)	11 (2, 20)	0.021
Hypertensive disorders in pregnancy^e^, no. (%)	21 (13.7)	39 (24.8)	− 11 (− 20, − 2)	9 (2, 16)	0.012

*CPAP* continuous positive airway pressure; *95%CI* 95% confidence interval

^a^Binary logistic regression analysis was used to calculate the risk difference and number needed to treat of preeclampsia and pregnancy induced hypertension between participants in CPAP versus usual-care groups

^b^Severe preeclampsia was defined according to Report of the American College of Obstetricians and Gynecologists’ Task Force on hypertension in pregnancy [31, 32]

^c^Early-onset preeclampsia was defined as developing preeclampsia before 34 completed weeks’ gestation

^d^Late-onset preeclampsia was defined as developing preeclampsia at or beyond 34 weeks’ gestation

^e^Hypertensive disorders in pregnancy comprised of preeclampsia and gestational hypertension

There were no significant differences in the other secondary outcomes (Additional file 2: Table S4). Serious adverse events were not reported. Rhinitis was most commonly reported in the CPAP and usual-care groups (17.7% vs 14%, p = 0.38) (Additional file 2: Table S5).

### Post-hoc analyses

#### Per-protocol and counterfactual analyses

For the per-protocol analysis, 50 participants with good adherence (average-CPAP use ≥ 4 h/night) in the CPAP-group and 155 participants in the usual-care group (excluding 2 participants who accidentally received CPAP during their 2nd trimester due to administrative error) were included. For the counterfactual approach, 52 participants who actually received and adhered to CPAP were compared to 258 participants who received no CPAP in the usual-care group or were CPAP-non-adherent (average-CPAP use < 4 h/nights).

Results for the per-protocol analysis also showed significant findings with mean differences for SBP, DBP, and MAP of − 3.8 (− 7.4, − 0.1), − 3.2 (− 5.7, − 0.8), and − 3.4 (− 6.1, − 0.7) mmHg respectively (Additional file 2: Table S6). Likewise, the counterfactual analyses showed significant findings with mean differences for SBP, DBP, and MAP of − 5.7 (− 13.8, 2.4), − 6.4 (− 11.8, − 0.9), and − 6.2 (− 12.2, − 0.1) mmHg (Additional file 2: Table S6). Data comparing results between CPAP-adherent (average-CPAP use ≥ 4 h/night), CPAP-non-adherent (average-CPAP use < 4 h/night) and usual-care groups also showed significant reduction in blood pressures in the CPAP-adherent subgroups as shown in Table 6.

**Table 6:** Explorative comparison of treatment effects between usual-care, CPAP non-adherent (average-CPAP use < 4 h/night), and CPAP-adherent (average-CPAP use ≥ 4 h/night) subgroups

	Usual-care (n = 157)	CPAP non-adherent subgroup (n = 103)	CPAP-adherent subgroup (n = 50)	*p *value
Treatment effect				
Overall mean blood pressure (SE) after randomization				
Systolic blood pressure, mmHg	116.07 (0.92)	115.11 (1.16)	112.22 (1.64)	
∆Overall difference^a^	Reference	− 0.96 (− 3.87, 1.95)	− 3.85* (− 7.54, − 0.14)	0.041
∆Adjusted overall difference^b^	Reference	− 0.83 (− 3.43, 1.77)	− 4.38* (− 7.68, − 1.08)	0.009
Diastolic blood pressure, mmHg	72.50 (0.61)	70.89 (0.77)	69.28 (1.08)	
∆Overall difference^a^	Reference	− 1.61 (− 3.53, 0.305)	− 3.23* (− 5.66, − 0.79)	0.009
∆Adjusted overall difference^b^	Reference	− 1.48 (− 3.11, 0.16)	− 3.54* (− 5.62, − 1.46)	0.009
Mean arterial blood pressure, mmHg	87.03 (0.68)	85.64 (0.86)	83.59 (1.21)	
∆Overall difference^a^	Reference	− 1.39 (− 3.54, 0.75)	− 3.44* (− 6.16, − 0.71)	0.013
∆Adjusted overall difference^b^	Reference	− 1.26 (− 3.10, 0.59)	− 3.83* (− 6.17, − 1.49)	0.001

*CPAP* continuous positive airway pressure; *SE* standard error of mean

*Denotes statistical significance, p-value < 0.05 compared to usual-care subgroup

^a^Intergroup difference is calculated as change in CPAP non-adherent and CPAP-adherent subgroups compared to that usual-care group using longitudinal data analysis with mixed-effect model after randomization presented as mean difference and 95%confidence interval (95%CI)

^b^Intergroup difference is calculated as change in CPAP non-adherent and CPAP-adherent subgroups compared to that usual-care group using longitudinal data analysis with mixed-effect model after randomization presented as mean difference and 95%confidence interval (95%CI) adjusted with chronic hypertension status, and anti-hypertensive medication use, and gestational age

Reductions in preeclampsia and hypertensive disorders in pregnancy were significant in the counterfactual approach, while only late-onset preeclampsia was significantly reduced in the per-protocol analysis (Table 7).

**Table 7:** Secondary outcomes on preeclampsia, and hypertensive disorders in pregnancy using the per-protocol and counterfactual analyses

Endpoints	CPAP group	Usual-care group	Risk difference, % (95%CI)^a^	Number needed to treat (95%CI)^a^	*p *value
*Per*-*protocol analysis*	(*n* = *50*)	(*n* = *155*)			
Preeclampsia, no. (%)	6 (12.0)	35 (22.6)	− 11 (− 22, 6)	–	0.124
Severe preeclampsia^b^	6 (12.0)	22 (14.19)	− 2 (− 8, 13)	–	0.684
Early preeclampsia^c^	4 (8.0)	4 (2.58)	5 (− 13, 3)	–	0.180
Late preeclampsia^d^	2 (4.0)	31 (20.0)	− 16 (− 24, − 8)	7 (3, 10)	0.024
Hypertensive disorders in pregnancy^e^, no. (%)	7(14.0)	39(25.2)	− 11 (− 23, 1)	–	0.120
*Counterfactual analysis*	(*n* = *52*)	(*n* = *258*)			
Preeclampsia, no. (%)	6 (11.5)	49 (19.0)	− 17 (− 27, − 6)	6 (2, 10)	< 0.001
Severe preeclampsia^b^	6 (11.54)	29 (11.24)	9 (− 19, 0.4)	–	0.062
Early preeclampsia^c^	4 (7.69)	4 (1.55)	2 (− 5, 5)	–	0.938
Late preeclampsia^d^	2 (3.85)	45 (17.44)	− 18 (− 27, − 10)	6 (3, 8)	< 0.001
Hypertensive disorders in pregnancy^e^, no. (%)^e^	7 (13.5)	53 (20.5)	− 19 (− 30, − 9)	5 (2, 8)	< 0.001

*CPAP* continuous positive airway pressure; *95%CI* 95% confidence interval

^a^Binary logistic regression analysis was used to calculate the risk difference and number needed to treat of preeclampsia and hypertensive disorders in pregnancy between participants in CPAP versus usual-care groups

^b^Severe preeclampsia was defined according to Report of the American College of Obstetricians and Gynecologists’ Task Force on hypertension in pregnancy [31, 32]

^c^Early-onset preeclampsia was defined as developing preeclampsia before 34 completed weeks’ gestation;

^d^Late-onset preeclampsia was defined as developing preeclampsia a or beyond 34 weeks’ gestation

^e^Hypertensive disorders in pregnancy comprised of preeclampsia and gestational hypertension

#### Subgroup analysis for mild OSA/upper airway resistance syndrome (UARS) and OSA

Although all participants had RDI ≥ 5 events/hour, those with AHI < 5 were classified as mild OSA/UARS and those with AHI ≥ 5 events/hour were classified as OSA [30, 37]. Exploratory analyses by these subgroups showed similar results as the main findings. Both subgroups respectively showed significant reductions in DBP (− 2.63 and − 2.11 mmHg), preeclampsia and hypertensive disorders in pregnancy on the adjusted modified-intention-to-treat analysis (Additional file 2: Tables S7, 8).

#### Subgroup analysis excluding participants randomized during 2nd trimester

Analyses excluding participants with new-onset OSA randomized during the 2nd-trimester also demonstrated significant results for CPAP treatment on reductions of BP and incidence of hypertensive disorders in pregnancy (Additional file 2: Tables S9, 10). None of the participants randomized during the 2nd-trimester in either group developed hypertensive disorders in pregnancy.

## Discussion

We conducted an RCT of high-risk pregnant women with mild/moderate OSA (RDI IQR 5–29.8 and AHI IQR 4–13) to assess the efficacy of CPAP in reducing BP and gestational hypertensive disorders. Our findings indicate that CPAP significantly reduced BP, with larger effects on DBP and MAP than SBP. Results were robust for all approaches including modified-intention-to-treat, per-protocol, and counterfactual approaches. In addition, CPAP treatment also significantly reduced preeclampsia and hypertensive disorders in pregnancy by 9% and 11%, respectively.

Although modified-intention-to-treat analysis is claimed as the least biased because it preserves random allocations, it may underestimate the causal-treatment effect because of dilution of noncompliers [36]. The counterfactual approach by instrumental variable analysis is an unbiased alternative that has been applied in previous RCTs [27, 38–41] when adherence to treatment is suboptimal, to estimate a complier-averaged treatment effect. All participants were used in this analysis considering initial randomization as the instrumental variable and treatment compliance as the exposure whereas the per-protocol analysis considered only participants who complied with the CPAP; in the latter, the randomization may not be guaranteed, leading to selection bias [36].

DBP significantly affects uteroplacental hemodynamics and is more strongly associated with the risk of preeclampsia than SBP [42–44]. Elevated DBP as pregnancy progresses is associated with the risk of preeclampsia, implicating the development of placental dysfunction [45, 46]. Although modest in magnitude, the observed changes in BP from our study are within the range of variability previously described but usually detected before the actual diagnosis of preeclampsia [46]. Although the BP reduction of 2 mmHg is modest and, this reduction is practically equivalent to the level found in the general population with OSA [34], it appears to be clinically beneficial given the concomitant reduction in the burden of preeclampsia.

Moreover, the true clinical effect may be larger with higher adherence to CPAP during pregnancy. Of note, this BP-lowering effect of CPAP was seen in both subgroups of participants, i.e. those who did and did not take anti-hypertensive medications. Fewer anti-hypertensive medications were required among those taking anti-hypertensive medications in the CPAP group (Table 3).

BP changes in our participants demonstrated the well-described pattern of mid-pregnancy fall, regardless of intervention groups [47]. Longitudinally, CPAP modified the physiological progression of BP by lowering DBP and MAP compared to early pregnancy values across all three trimesters. In contrast, BP in the usual-care group followed the physiological changes with a decrease only during the mid-pregnancy period. Normal hemodynamic adaptation before the mid-pregnancy nadir includes decreased total vascular resistance which benefits placental perfusion [47, 48]. BP increases from this point forward reflect the increasing production of vasoconstrictive agents from the growing placenta, and the increasing cardiovascular demands of pregnancy [48]. Hypoperfusion of the placenta induces production of vasoconstrictive agents often found in women who developed preeclampsia [49]. The proposed sequelae of placental hypoperfusion include systemic endothelial dysfunction via the release of anti-angiogenic agents (e.g. the soluble fms-like tyrosine kinase-1, sFlt-1) [49]. Recently, a case report demonstrated that CPAP treatment could control OSA and sFlt-1 concentrations in a high-risk pregnancy with chronic hypertension, supporting the possible link between placental hypoxia and endothelial dysfunction which may progress to preeclampsia [50]. The reductions of DBP, and the modulation of BP trajectory from our study suggest that CPAP treatment may have an effect on placental physiology, but this needs to be explored in other studies.

Preeclampsia is a heterogeneous disorder involving multiple placental mechanisms ranging from poor implantation to placental stress and hypoxia caused by “abnormal placentation” as in early-onset preeclampsia or “maternal stress factors” as in late-onset preeclampsia [51, 52]. In this study CPAP was effective only in late-onset, but not early-onset preeclampsia; this could be explained by low power, the later initiation of CPAP exceeding the critical period of placentation (0–13 weeks) [51, 52], or inadequate auto-CPAP titration [53]. However, whether or not maternal OSA and its treatment alter the process of placental implantation is unknown. Maternal OSA has been associated with histopathological evidence of fetoplacental hypoxia, and placental overgrowth [14, 15]. Preconception screening and treatment of OSA should be further studied.

Our findings replicated Guilleminault’s studies which similarly showed that the BP-lowering effect of CPAP was markedly apparent after 6 months of gestation, with more anti-hypertensive medication requirement in controls, and more favorable pregnancy outcomes in the CPAP-group [26, 54]. These studies included hypertensive pregnant women with only subtle sleep-disordered breathing (i.e., snoring, airflow limitation or RDI ≥ 3 events/hour) [26, 54]. The previous RCT participants all had AHI < 5 events/hour with a mean of only 3.1 [26]. A recent non-randomized prospective cohort of high-risk pregnancy with mild/moderate OSA (median respiratory event index, REI 5.5 events/hour) treated with CPAP for 4 weeks also showed reductions in incidence of preeclampsia [24]. Another real-world retrospective study in high-risk pregnant women with moderate OSA (mean REI 17.5 events/hour) treated with CPAP also demonstrated similar results in reducing hypertensive disorders in pregnancy [25].

Recent data indicated that airflow limitation or RERA could cause an increase in BP similar to apnea/hypopnea in pregnant population [19, 55]. Thus, our study included mild OSA based on RDI rather than AHI. Our findings also demonstrated that subgroups with AHI < 5 but RDI ≥ 5, variably referred to as “mild OSA” versus “UARS” might still benefit from CPAP treatment in lowering DBP, preeclampsia, and hypertensive disorders in pregnancy, indicating that AHI alone may not sufficiently capture clinically significant OSA during pregnancy. This signifies that in addition to conventionally-designated moderate to severe OSA, high-risk pregnancy with “mild OSA” or “UARS” may still benefit from CPAP treatment. Although in our study, frequent snoring was reported in half of our participants and listed as one of the inclusion criteria, eligibility was based entirely on objective RDI criteria and patients who did not snore also had RDI ≥ 5.

One strength of our trial was that full-night polysomnography was performed on all participants to detect both early-pregnancy and new-onset OSA. Due to different exposure time, early-pregnancy OSA (or probably chronic longstanding OSA) and new-onset OSA may be clinically different [9]. As 96% of the participants had early-pregnancy OSA, sensitivity analysis of this subgroup excluding the new-onset OSA showed similar results to the main study. However, there was limitation for further analysis for the new-onset OSA due to the small number of participants and absence of hypertensive disorders in pregnancy in this subgroup. Further studies of this latter group may be needed.

The high OSA detection rate in our study may not reflect the true OSA prevalence of this population because only 385 (35.1%) of the total 1098 high-risk pregnant women agreed to participate for polysomnography. There might be a selection bias in that those who had more symptoms might have been more motivated to participate. Furthermore, the highly sensitive diagnosis with polysomnography and RDI criteria was used in a high-risk pregnancy population. Based on the home-sleep-apnea test (AHI ≥ 5 criteria), previous studies found OSA prevalence in high-risk pregnancy during 1st and 3rd trimesters of approximately 28–60.3% and 24–50%, respectively [4–7, 56]. However, data on the prevalence of OSA using RDI ≥ 5 criterion in high-risk pregnancy were lacking.

The main limitation of our trial was CPAP nonadherence, which occurred in a large percentage of participants, comparable to other RCTs [27, 28, 57, 58]. CPAP treatment for mild/moderate OSA can be challenging, with reported low acceptance rates of 10–37.4% [27, 28, 57, 58]. Although our sample size did not account for nonadherence, per-protocol and counterfactual analyses demonstrated greater reductions in BP and preeclampsia in participants with higher adherence/average hours of CPAP treatment [36]. Significant reduction in BPs was also shown in the CPAP-adherent subgroups (average-CPAP use ≥ 4 h/night) when compared to CPAP-non-adherent (average-CPAP use < 4 h/night) and usual-care groups. Given that pregnancy is a short period for adaptation to CPAP use, further study on the pattern of CPAP use and measures to improve adherence should be pursued.

Our results may not be generalizable to pregnant women without pre-defined high-risk factors or those with severe OSA. Despite successful treatment case reports, the magnitude of CPAP treatment effect on severe cases is unknown [59]. We caution that results of early CPAP treatment, as in our study, may not reflect CPAP treatment during late pregnancy or when preeclampsia has already occurred. Differences in other maternal–fetal outcomes could not be detected because of lack of power.

In conclusion, evidence from a multicenter RCT of high-risk pregnancies with mild/moderate OSA indicates that early CPAP treatment significantly lowers DBP, MAP, and reduces the incidence of preeclampsia and hypertensive disorders in pregnancy. This raises the need for early diagnosis and treatment of OSA in high-risk pregnancies.

## Supplementary Information

**Additional file 1.** Study protocol.

**Additional file 2.** Additional.

## Data Availability

Available from the corresponding author on reasonable request.

## Abbreviations:

AHI: Apnea–hypopnea index
Auto-CPAP: Auto-adjusting CPAP
BP: Blood pressure
BMI: Body mass index
CPAP: Continuous positive airway pressure
DBP: Diastolic blood pressure
GA: Gestational age
GDM: Gestational diabetes
IQR: Interquartile range
OSA: Obstructive sleep apnea
RCT: Randomized controlled trial
RDI: Respiratory disturbance index
SD: Standard deviation
SBP: Systolic blood pressure
