# Supplementary material for: Treatment of obstructive sleep apnea in high risk pregnancy: a multicenter randomized controlled trial
Source: Respir Res. 2023 Jun 27;24:171. doi: 10.1186/s12931-023-02445-y (PMC10294320; doi:10.1186/s12931-023-02445-y)
Supplement: Supplementary file 1 — Additional file 1. Study protocol. [file 12931_2023_2445_MOESM1_ESM.pdf]

Screening and treatment of obstructive sleep apnea in high-risk pregnancy: A multicenter randomized controlled

Final version 6/10/2020

NCT number: ClinicalTrial.Gov NCT03356106

Registered 11/22/2017

1st Ethics approval 2/9/2016

1st Ethics amendment 12/27/2018

Final version amendment 6/10/2020

Screening and treatment of obstructive sleep apnea in high-risk pregnancy: A multicenter randomized controlled

Final version amendment 6/10/2020

1.1 Background and rationale

1.1.1 Magnitude of problem:

Obstructive sleep apnea (OSA) is caused by the cyclical upper airway collapses during sleep resulting in arousals, sleep fragmentation, intermittent oxygen desaturation, and sympathetic activation [1]. The prevalence of OSA in general population varied from approximately 2% to 26% worldwide [2, 3]. Prevalence of OSA in general population approximates 4% in men, and 2% in women [2]. Similarly, the prevalence of OSA in Thai population approximates 4.3-4.4% (5.3-15% male; 1.9-3.9% female) [4, 5]. However, it has been estimated that up to 93% of women and 82% of men with moderate to severe OSA have still remained undiagnosed [6]. The prevalence of sleep disordered breathing (SDB) in high-risk pregnancy was also high, which ranged from 20% to 35% (16-21) as for diagnosis based on objective sleep tests (e.g., polysomnography (PSG) or Watch-PAT) [7-12]. Recent meta-analysis showed the pooled prevalence of SDB during pregnancy of 26.7% (95%CI: 16.9%, 34.4%, I^2=83.15%) [13]. However, prevalence of SDB in pregnancy remains uncertain depending on various factors such as population studied (high vs low risk), type of diagnostic measures, and trimester of study.

1.1.2 Disease burden and impacts

Growing evidences indicated that sleep disordered breathing during pregnancy is associated with poor maternal and fetal outcomes, e.g., gestational hypertension (GHT), pre-eclampsia, gestational diabetes (GDM)and low infant birth weight as for evidences from meta-analyses [14-16]. This might be due to physiologic and hormonal changes in pregnancy and thus predispose towards development of new-onset or exacerbation of SDB [17].

However, diagnosis and screening of SDB during pregnancy are challenging. Although in-laboratory PSG is the gold standard diagnostic test, long waiting periods for appointments, particularly considering the short time-window to perform the test early during the pregnancy, and the discomfort induced by the sleep test may lead to absence of investigation [18, 19].

Thus, simple and accurate screening strategies should be investigated. Risk stratification for the probability of OSA in pregnant women will also help in prioritizing the need for further diagnostic sleep testing given the limited resources in many places throughout the world. Early diagnosis and treatment of OSA in pregnancy should be implemented given the potential benefit on pregnancy outcomes [20, 21].

Our recent systematic review and meta-analysis showed that conventional OSA screening questionnaire such as the Berlin questionnaire and Epworth sleepiness scale had poor performance during pregnancy. We conclude that the poor discriminative values might be related to the facts that 1) both OSA and pregnancy lead to similar sleep complaints; 2) that there is a continuous change in symptomatology and the severity of the sleep-disordered-breathing with progression of pregnancy; 3) that the standard and threshold to diagnose SDB during pregnancy has not been defined; 4) finally that the timing of the questionnaire administration is not standardized.

And there is a need for screening tool that take into account the change overtime of the symptoms of OSA, and pregnancy. New screening tools and strategies specific to pregnancy that enable us to sequentially screen and monitor for OSA throughout pregnancy should be developed.

1.1.3 Treatment & goal of treatments

Nasal continuous positive airway pressure (CPAP), the standard treatment for OSA in non-pregnant population. CPAP has been shown to reduce cardiovascular mortality, morbidity, and blood pressure [22-25]. However, there are limited data regarding the efficacy of CPAP treatment in pregnancy. CPAP also has been proposed as the adjunct therapy for pre-eclampsia [26]. Despite the safety use of CPAP in pregnant women, the results regarding improvement of pregnancy outcome were lacking [27]. Extrapolation of treatment effect of CPAP from other population may not apply to pregnancy given a different physiology during pregnancy and the effect on the fetus.

We performed a systematic review in search of the existing studies concerning the efficacy of CPAP treatment for SDB during pregnancy. Of the 7 studies included (n=111), only 3 studies were randomized controlled trials (RCT), and the other 4 studies were experimental studies with pre-post design. And of the 3 RCTs, only 1 study initiated CPAP during 1st trimester and throughout pregnancy, the other 2 RCTs applied CPAP for only 1 night.

Significant results for CPAP efficacy regarding BP-lowering effects were reported in 2 studies with the early pregnancy and long-term use, and 2 studies with 1-night use of CPAP in the pre-eclamptic women during 3rd trimester, while 1 study in gestational hypertension women with 1-night use of CPAP showed no treatment effect.

Data on pregnancy outcomes were reported in the 3 long-term early CPAP use studies. While CPAP use seemed to prevent the incidence of preeclampsia, and preterm in pregnant women with chronic HT, this was not the case in women with preeclampsia risk and obesity.

Based on the rarity of evidence, it is too early to make any conclusion given the limitation in the sample size of the studies, the difference in study design, outcome measurement, the timing and duration of CPAP administration, and characters of subjects included. However, there are some important conclusions that we can make. First, despite the association of SDB and poor maternal and fetal outcomes shown in meta-analyses, there is not enough evidence from existing studies that CPAP treatment can improve pregnancy outcomes particularly, the reduction of preeclampsia and preterm labor. Second, there is no definite cut-off threshold for SDB diagnosis during pregnancy. Third, early and long-term CPAP treatment initiating during 1st trimester seemed more promising than in late pregnancy in improvement of pregnancy outcome. And lastly, in pre-eclamptic women during late pregnancy there is no evidence that CPAP can modify the disease progression, although 1-night CPAP might lower nocturnal blood pressure.

Hence, SDB pose a significant threat to pregnancy and fetus, diagnosis and treatment of SDB during pregnancy are still challenging given the dynamic changes of pregnancy and SDB. Because of the lack of evidence for the diagnostic and treatment threshold for SDB during pregnancy, there is currently no standard guideline to systematically screen and treat for SDB during pregnancy. The situation of SDB during pregnancy is in state of "clinical equipoise", where the scientific community needs a strong evidence to make any treatment recommendation for the care of pregnant women at risk. It is an urgent need to conduct a randomized controlled trial that can demonstrate the efficacy of CPAP treatment in high risk pregnant women.

1.2 Research Question

1.2.1 Does the use of CPAP treatment for SDB in pregnant women with preeclampsia risk result in better blood pressure control and thus reduce related maternal and fetal complications than standard usual antenatal care in control group

1.2.2 We want to investigate whether our proposed screening algorithm for SDB specific to pregnancy is accurate and feasible in monitoring and diagnosis of SDB during pregnancy.

1.3 Research Objectives

1.3.1 **Primary Objective**: To compare whether the treatment effect of CPAP on SDB during pregnancy can improve blood pressure control in high risk pregnancy

- Systolic/diastolic blood pressure during daytime ANC at 10:00-12:00 am at randomization (GA 0-16 week), 1.5-2 month after randomization (GA 18-20 week), and 3-4 month after randomization (GA 24-28 week), 5-6 month after randomization (GA 32-34 week) and monthly (GA>35 week) until delivery

1.3.2 **Secondary Objective:**

- To compare the composite outcome of pre-eclampsia/GHT, GDM, fetal growth restriction, and pre-term labor between the CPAP treatment and control groups. And individual outcomes.
- To compare the night-time blood pressure compared to control group. 2nd outcome

2.1 Definitions

Obstructive sleep apnea (OSA)/Sleep disordered breathing (SDB)/Upper airway resistance syndrome (UARS): Diagnostic criteria according to the International Classification of Sleep Disorders. 3rd Edition includes (A and B) or C satisfy the criteria [28]

A. The presence of 1 or more of the following:

    1. The patients complain of sleepiness, nonrestorative sleep, fatigue, or insomnia symptom
    2. The patient wakes with breath holding, gasping, or choking
    3. The bed partner or other observer reports habitual snoring, breathing interruptions, or both during the patient's sleep.
    4. The patient has been diagnosed with hypertension, a mood disorder, cognitive dysfunction, coronary artery disease, stroke, congestive heart failure, atrial fibrillation, or type 2 diabetes mellitus

B. PSG or out-of-center sleep test (OCST) demonstrates:

    1. 5 or more predominantly obstructive respiratory events (obstructive and mixed apneas, hypopneas, or respiratory effort related arousal (RERAs) per hour of sleep during a PSG or per hour of monitoring (OSCT).

OR

C. PSG or OCST demonstrates:

1. 15 or more predominantly obstructive respiratory events (obstructive and mixed apneas, hypopneas, or respiratory effort related arousal (RERAs) per hour of sleep during a PSG or per hour of monitoring (OSCT).

Apnea: a reduction of thermistor signal at least 90% from baseline lasting for at least 10 seconds. Obstructive apnea is classified by the presence of RIP effort, and central apnea is classified by the absence of RIP effort [18].

Hypopnea: a reduction in nasal pressure transducer signal at least 30% from baseline that is associated with either O2 desaturation ≥ 3% from baseline or EEG arousal.

Respiratory effort related arousals (RERA): a sequence of breaths lasting ≥ 10 seconds characterized by increasing respiratory effort or by flattening of the inspiratory portion of the nasal pressure leading to arousal from sleep that does not meet the criteria for apnea or hypopnea.

Apnea-hypopnea index (AHI): = no. of apnea+hypopnea events/Total sleep time (hr)

Respiratory disturbance index (RDI): no. of apnea+hypopnea+RERA/Total sleep time (hr)

Hypertensive disorder of pregnancy consisted of 4 categories [29]

a) **Preeclampsia-eclampsia:** diagnostic criteria includes

- **Blood pressure:**
    - ≥ 140/90 mmHg on 2 occasions at least 4 hours apart after 20 weeks of gestation in a woman with a previously normal blood pressure
    - ≥ 160/110 mmHg, hypertension can be confirmed within a short interval (minutes) to facilitate timely antihypertensive therapy

And

- **Proteinuria**

- ≥ 300 mg per 24-hour urine collection (or this amount extrapolated from a timed collection) OR
- Protein/creatinine ratio ≥ 0.3 (each measured as mg/dL)
- Dipstick reading of 1+ (used only if other quantitative methods not available)

Or in the absence of proteinuria, new-onset hypertension with the new onset of any of the following:

- **Thrombocytopenia**
  - Platelet count <100,000/microliter
- **Renal insufficiency**
  - Serum creatinine concentration > 1.1 mg/dL or a doubling of the serum creatinine concentration in the absence of other renal disease
- **Impaired liver function**
  - Elevated blood concentrations of liver transminases to twice normal concentration
- **Pulmonary edema**
- **Cerebral or visual symptoms**

b) **Chronic hypertension (of any cause) is** hypertension that predates pregnancy

c) **Chronic hypertension with superimposed preeclampsia** is chronic hypertension in association with preeclampsia

d) **Gestational hypertension** is BP elevation after 20 weeks of gestation in the absence of proteinuria or the aforementioned systemic findings.

Severe preeclampsia is defined with any of these findings:

- ≥ 160/110 mmHg on 2 occasions at least 4 hours apart while patient is on bed rest (unless antihypertensive therapy is initiated before this time)
- Thrombocytopenia (platelet counts <100,000/microliter)
- Impaired liver function as indicated by abnormally elevated blood concentrations of liver enzymes (to twice normal concentration), severe persistent right upper quadrant or epigastric pain unresponsive to medication and not accounted for by alternative diagnoses, or both
- Progressive renal insufficiency (serum creatinine concentration > 1.1 mg/dL or a doubling of the serum creatinine concentration in the absence of other renal disease)
- Pulmonary edema
- New-onset cerebral or visual disturbance

Gestation diabetes is defined as diabetes diagnosed in the second or third trimester of pregnancy that is not clearly overt diabetes [30]. Criteria are listed below

- **One-step strategy:** 75-g OGTT when patient is fasting and at 1-hour and 2-hour at 24-28 weeks of gestation in women not previously diagnosed with overt diabetes. GDM diagnosis is made with any of the following plasma glucose met or exceeded:
    - Fasting 92 mg/dL
    - 1-hour 180 mg/dL
    - 2-hour 153 mg/dL

- **Two-step strategy**

  - **Step 1:** perform 50-g GLT (nonfasting), with plasma glucose measurement at 1 h, at 24-28 weeks of gestation. If plasma glucose level at 1 hour after the load is 140 mg/dL, proceed to a 100-gm OGTT.

  - **Step 2:** the 100-g OGTT should be performed when patient is fasting. The diagnosis of GDM is made if at least 2 of the following 4 plasma glucose levels are met or exceeded**:**

Carpenter/Coustan

- Fasting 95 mg/dL
- 1-hour 180 mg/dL
- 2-hour 155 mg/dL
- 3-hour 140 mg/dL

HbA1C, fasting plasma glucose, 1-hour plasma glucose, 2-hour plasma glucose had been shown to be predictor of preeclampsia.

Fetal growth restriction is defined as a customized birthweight <10th centile for gestational age [31].

Preterm labor is defined as is the birth of a baby at less than 37 weeks gestational age [31, 32]

2.2 Conceptual framework

Conceptual framework is shown in figure 1

3. METHODOLOGY

3.1 Study design and setting

The study design will be a multicenter randomized-controlled trial in 3 tertiary care hospitals including Ramathibodi Hospital, Phramongkutklao Hospital and Rajavithi Hospital.

3.1.1 Ramathibodi Hospital is a university hospital of the Faculty of Medicine Ramathibodi Hospital which is a faculty within Mahidol University and the fourth oldest medical school in Thailand. The hospital serves at least 5,000 out-patient visits per day and in-patients with more than 1,000 beds for tertiary medical care. The hospital provides approximately 3,000 antenatal care service and delivery each year. It will be the primary site of the research investigation. The study will initiate here in June 2016 and followed by other centers. The study will take approximately 2 years for the accrual time to achieve the target number of participants and another year for the completion and analysis.

3.1.2 Phramongkutklao College of Medicine is the first and only medical cadet school in Thailand. The college is the seventh medical school established in Thailand by the will of King Bhumibhol Adulyadej. Phramongkutklao College of Medicine is an affiliated college of Mahidol University. It is the teaching hospital accommodating 1,200-bed in the tertiary level. The hospital provides approximately 3,000 antenatal care service and delivery each year. The research will start soon after the study initiation in Ramathibodi Hospital once the ethics committee and funding are approved. The study will take approximately 2 years for the accrual time to achieve the target number of participants and another year for the completion and analysis.

3.1.3 Rajavithi Hospital is a 1,200-bed medical center accommodating 40,000 in-patients and 1,000,000 out-patients yearly or a daily average of 4,000 patients, with 200,000 referral patients (both in and out patients). The hospital provides medical service at standard health promotion in tertiary levels and above, including quality referral system. The hospital provides approximately 4,000 antenatal care service and delivery each year. The research will start soon after the study initiation in Ramathibodi Hospital once the ethics committee and funding are approved. The study will take approximately 2 years for the accrual time to achieve the target number of participants and another year for the completion and analysis.

3.2 Study subjects

High risk pregnant women attending the antenatal care at the collaborated hospitals will be eligible for the study if they meet all following inclusion criteria.

3.2.1 Inclusion criteria (ALL criteria)

- Singleton pregnant women age ≥ 18 years without significant medical conditions such as immunocompromised status, chronic infection (HIV infection, tuberculosis), chronic lung and cardiac conditions, chronic kidney disease, thyroid disease, or neuromuscular disease.
- Pregnant women attending the 1st antenatal care and intending to deliver at one of the study hospitals with gestational age < 16 weeks
- Thai nationality with ability to use Thai language proficiently both in spoken and written language.
- Have been diagnosed as high-risk pregnancy by obstetricians by having any of the following conditions

a. Chronic hypertension prior to pregnancy

b. Hypertension during pregnancy before 20week gestation.

c. Pre-eclampsia/gestational hypertension during recent pregnancy

d. Obesity defined by pre-pregnancy body mass index (BMI) ≥ 27.5 kg/m^2

e. Having diabetes mellitus or history of gestational diabetes in previous pregnancy

- Having SDB by either definition
    a) RDI ≥ 5 events/hour by sleep testing at either point of testing during early pregnancy (0-16 GA week) or late pregnancy (GA 24-28 week)
    b) OR Presence of snoring (either as frequent snoring ≥3 times/week or loud snoring) prior to or occurring during pregnancy
- Sign written informed consent

3.2.2 Exclusion criteria

- Subjects with severe obstructive sleep apnea (RDI ≥30 events/hour) or with significant O2 desaturation <80% during sleep that might have potential benefit from the CPAP treatment. CPAP treatment will be offered to these subjects.
- Subject with known obstructive sleep apnea and currently on CPAP treatment

Recruitment of subjects will be done at the antenatal care clinic. Screening for eligibility by investigators will be done, consecutively. Subjects who meet the inclusion criteria without any of the exclusion criteria will be approached to participate in the study.

3.3 Randomization and allocation

A randomization list will be generated and stratified by trimesters. For each stratum, a block randomization with varying block size from 4 to 8.

Preparation of randomization will be done by a third party, not involved in the clinical trial. Randomization sequence codes were generated using centralized computer system. Once eligible participants had agreed to participate the study and signed informed consent, the investigators entered clinical details into the system. Then the computer system generated the automatic randomization sequence and immediately sent back to the investigators for further allocation accordingly. After randomization and allocation, participants in the CPAP intervention group received CPAP treatment nightly throughout pregnancy until delivery.

3.4 Blinding

This is an open-label randomized controlled trial, in which subjects will know each intervention she has received between CPAP machine and standard usual antenatal care. A blinded evaluation is planned to assess the observed primary and secondary end points by a separate outcome assessor.

3.5 Variable & measurement

3.5.1 Polysomnography (PSG)

Screening for sleep disordered breathing (SDB) or obstructive sleep apnea (OSA) with either in-laboratory or home PSG will be performed depending on subject's preference. Performance of polysomnography will be done with SomnoTouchResp, which consisted of

- channels of electroencephalographies (EEG: F4M1, C4M1, O2M1)
- channels of electro-oculographies (EOG: ROC, LOC)
- Chin electromyography (EMG)
- Respiratory signals include nasal pressure transducer, oronasal thermistor, and pulse oximeter

- respiratory efforts using thoracic and abdominal respiratory inductance plethysmography (RIP)
- Sleep staging and respiratory scoring are according to the American Academy of Sleep Medicine 2012 version 2.2 [18]

    1). Apnea is defined as a reduction of thermistor signal at least 90% from baseline lasting for at least 10 seconds. Obstructive apnea is classified by the presence of RIP effort, and central apnea is classified by the absence of RIP effort.

    2). Hypopnea is defined as a reduction in nasal pressure transducer signal at least 30% from baseline that is associated with either O2 desaturation ≥ 3% from baseline or EEG arousal.

    Apnea-hypopnea index is calculated as numbers of respiratory events per hour of total sleep time or recorded time.

    3). Respiratory effort related arousal (RERA) is defined as a sequence of breaths lasting ≥ 10 seconds characterized by increasing respiratory effort or by flattening of the inspiratory portion of the nasal pressure leading to arousal from sleep that does not meet the criteria for apnea or hypopnea.

    4). Additional scoring of airflow limitation and snoring time will be included as previously described [33].

Tabulation of respiratory index into apnea-hypopnea index [AHI] = no. of apnea+hypopnea/Total sleep time (hr)] and respiratory disturbance index [RDI =no. of apnea+hypopnea+RERA/Total sleep time (hr)] will be done.

3.5.2 Measurement of blood pressure

- Measurement of blood pressure using manual sphygmomanometer during ANC visit will be done twice by research nurse in sitting resting position on both arms at least 15 minutes apart. Mean of the 4 values will used for the analysis.
- 24-hour blood pressure monitoring (ABPM) will be done
    - 24-hour BP monitoring device during daytime and night-time [34, 35] had been used in pregnancy population and showed good correlation with uterine artery Doppler ultrasound. “Non-dipping pattern” on the 24- hour blood pressure monitoring had been shown can be to associated with preeclampsia and small for gestational age [36-39]. Validation of particular ABPM device is required based on the criteria set by the European Society of Hypertension position paper on ambulatory blood pressure monitoring [35]. The 24-hour BP monitoring in early pregnancy (GA<14 weeks) has been shown to predict occurrence of preeclampsia [40]. Furthermore, 24-hour SBP (per 10 mmHg [odd ratio (OR): 1.74; 95%CI:1.28-2.38; p<0.001)] was more closely associated with small-for-gestational age (SGA) than clinic BP (OR 1.40;95%CI:0.92-2.13; p=0.11) [41].
    - Non-invasive blood pressure monitoring using the calculation from pulse transit time method (PTT). Validation of this device according to the European Society of Hypertension criteria had been done in non-pregnant population [42]. The device-observer disagreement was -0.44 6.1 mmHg for SBP and -0.33 3.4 mmHg for DBP. A strong correlation was observed between Somnotouch-NIBP and auscultatory method for SBP (r=0.973) and

for DBP (r=0.976). The BP discrepancies did not vary according to sex (p>0.15), age, body weight, arm circumference (p>0.10). But a significant correlation was found between SBP discrepancy and height (r=0.4) [42].

3.5.3 Fetal ultrasound and Doppler ultrasound.

Fetal ultrasound and Doppler ultrasound will be performed by experienced obstetrician to monitor parameters listed below [43]:

- Fetal growth by ultrasound: constant (above, below, or on curve); Falling off with increasing gestation (12 week, 18-20 week, 28 week, 36 week)
- Fetal hemodynamic including uterine, umbilical, and fetal middle cerebral artery blood flow indices at mid gestation (16-25 weeks) including 1) notching (yes/no): bilateral (yes/no); unilateral (yes/no); 2) pulsatility index (PI) and resistance index (RI). PI had been shown to be predictors of preeclampsia and intra-uterine fetal growth restriction [44, 45]. However, administration of methyl-dopa to lower blood pressure in preeclampsia showed no significant change after treatment [46-48].
- Amount of amniotic fluid.

3.5.4 Measurement of plasma glucose as described by 75-g OGTT. HbA1C, fasting plasma glucose, 1-hour plasma glucose, 2-hour plasma glucose had been shown to be predictor of preeclampsia with odd ratio of 1.42 (1.33-1.52), 1.46(1.37-1.55), 1.40(1.32-1.50), 1.37(1.29-1.46), respectively [49].

3.6 Data collection

3.6.1General management protocol for high risk women consisted of

3.6.1.1 During 1st trimester (First ANC, and subsequent 6-8 weeks ANC follow ups)

- Clinical data collection such as demographic data, underlying disease, number of gestations, last menstrual period, height, pre-pregnancy weight, pregnancy weight, neck circumference, general physical examination, and systolic and diastolic blood pressure.
- Laboratory including fasting HbA1C, fasting blood sugar, complete blood count, serologic blood test (rubella titer, anti-HIV, VDRL, Rh blood group),
- Fetal ultrasound, uterine artery Doppler

3.6.1.2 Second trimester

- Routine ultrasound at 18-20week gestation
- OGTT screening for GDM at 24-28week gestation
- 24-28week gestation: optional ultrasound depending on obstetrician's clinical decision
- Repeat blood test including fasting HbA1C, fasting blood sugar, complete blood count, and serologic blood test (anti-HIV, VDRL, Rh blood group)
- Urinary analysis

3.6.1.3 Third trimester

- Bi-weekly to weekly ANC follow-ups until delivery
- Ultrasound at 32-34week gestational age

3.7 STUDY INTERVENTION

3.7.1 CPAP intervention

- Immediately after allocation, subjects in the intervention arm will receive CPAP treatment throughout pregnancy.
- Auto CPAP machine will be prescribed to subjects with pressure in the range of 4-15 cmH2O. Optimal pressure to eliminate respiratory events will be achieved based on the 90-95 percentile pressure.
- After determination with auto-CPAP, the machine with optimal pressure (range 4-15 cmH2O) adjustment will be prescribed. Subjects will be encouraged to use CPAP every night, continuously. Daily record of applying CPAP will be provided with SD card within the machine, which including the start and stop time start in CPAP usage.
- During subsequent trimester when reaching 24-28 weeks of gestation, another adjustment for optimal CPAP pressure will be determined with the auto-CPAP machine. In case if higher CPAP pressure is required, adjustment will be made accordingly.
- Data of CPAP usage and adherence will be automatically recorded in the machine and available for download during follow-up.

3.7.2 Control Group

- Subjects will receive standard antenatal care and follow-up plan similar to the intervention group.

Primary outcome

- Systolic and diastolic blood pressure, which will be measured at rest using sphygmomanometer twice on both arms by research nurses during daytime ANC at 10:00-12:00 pm at randomization (GA 0-16 week), 1.5-2 month after randomization (GA 18-20 week), and 3-4 month after randomization (GA 24-28 week), 5-6 month after randomization (GA 32-34 week) and monthly (GA>35 week) until delivery

Secondary outcome

- 24-hour ambulatory blood pressure monitoring (daytime versus night time) using the non-invasive technique from pulse transit time (SomnoTouch NIBP).
- Pregnancy complication listed as composite endpoints below

(Composite endpoint): A combination of clinical endpoints in maternal and fetal outcomes, data from Ramathibodi hospital during 2013-2014) *

- <u>**Composite 1**: hypertensive disorder of pregnancy;</u>
- <u>**Composite 2:** hypertensive disorder of pregnancy or preterm labor or fetal growth restriction</u>
- **Composite 3:** Preterm labor or fetal growth restriction, or severe pre-eclampsia or eclampsia or death; (composite 3 incidence=10%)

*Given that the incidence of hypertensive disorder incidence=136/3787=3.6%; gestational diabetes incidence= 601/3787=15.9%; fetal growth restriction incidence= 157/3787=4.1%; preterm labor incidence=10%; and severe pre-eclampsia/eclampsia/ HEELP syndrome incidence= (43+ 1+3=46/3787=1.2%), respectively.

- Individual component of the composite endpoints including incidence of hypertensive disorder of pregnancy, gestational diabetes, preterm labor, and fetal growth restriction
- Glucose level, Oral Glucose Tolerance Test (OGTT), HbA1C
- Fetal ultrasound results: uterine Doppler arterial blood flow, fetal growth restriction
- Neonatal outcome: birth-weight, APGAR score, Length of stay, NICU admission

- Placenta character: weight will be measured at birth by the neonatal nurse.

3.8 Data collection

Schedule of data collection are shown in protocol flow chart and data table, see figure 2, and table 1

3.8.1 Job description of study personnel

Principle investigator will designate a specific job to the research team member. Job description according to each researcher will be clearly specified.

3.8.2 Training study personnel

Research assistants will be trained regarding to good clinical practice guideline, protocol, and collecting the data. Training of performing sleep test, and applying and retrieval of CPAP data will be held at Ramathibodi Hosptial Sleep Disorder Center**.**

3.8.3 Data collection and management

Case record form (CRF) will be developed to collect data regarding baseline demographic data, intervention, primary and secondary endpoints, adherence to interventions, and adverse effect.

3.9 Sample size estimation

3.9.1 Based on primary outcome

Power	Alpha	SD	Mean difference	M (Intervention /control)	Intervention N	Control N	Total N
0.80	0.05	7.4	2.0	1	216	216	432
0.80	0.05	7.4	2.5	1	139	139	278
0.80	0.05	7.4	3.0	1	111	111	222
0.80	0.05	7.4	3.5	1	71	71	142

Sample size calculation will be based on the primary outcome, which is the reduction in blood pressure. Based on the previous data by Reid, et al the mean diastolic blood pressure during pregnancy approximates 92.7±7.4mmHg [50]. Type I & II error and a ratio of intervention versus control are set at 0.05, 0.2, and 1:1, respectively. Estimated sample sizes are display below according to level of mean differences.

A systematic review and meta-analysis showed that CPAP could reduce blood pressure in OSA patients of -2.87 (95%CI -5.18-0.55) [25]. We, therefore, set mean difference of at least 2.5 mmHg, which will require 278 subjects to enroll. Taking into account for loss to follow-up of 20%, a total of 334 subjects are required (167 each arms). Additional number of subjects to be recruited was increased during January 2019-May 2019 up to 360 cases to allow for the potential subject discontinuation of the study with approval of the ethic committee.

3.9.2 Based on composite endpoint

Sample size calculation is also done according to the composite endpoint.

- **Composite 1 (**hypertensive disorder of pregnancy**)**

For composite endpoint 1, sample size of 134 each arm is required to detect difference of 15% with type I and II error of 0.05 and 0.20 respectively. Therefore, given the sample size of 153 each arm from the primary endpoints, it is possible to include this composite endpoint.

- **Composite 2 (**hypertensive disorder of pregnancy or preterm labor or fetal growth restriction or emergency C/S**)**

For composite endpoint 2, sample size of 141 each arm is required to detect the difference of 15% with type I and II error of 0.05 and 0.20 respectively. Therefore, given the sample size of 153 each arm from the primary endpoints, it is possible to include this composite endpoint.

And given the limitation in number of participant enrollment (n=306), estimation of the power of the study based on the effect size of the CPAP on composite endpoint 2 are done below. To achieve a difference of 12% and 15% on the composite endpoint 2, given the sample size of 306, it will achieve the power of 0.83 and 0.97, respectively.

- **Composite 3**

For composite endpoint 3, sample size of 492 each arm is required to detect difference of 6% with type I and II error of 0.05 and 0.20 respectively. Therefore, given the sample size of 153 each arm from the primary endpoints, it is **Not** possible to include this composite endpoint.

3.10 **Data analysis**

3.10.1Case record form (CRF)

CRF consisted of standardized forms to collect data regarding enrollment form, demographic data, follow-up data, polysomnographic data, CPAP adherence data, fetal ultrasound data and pregnancy outcomes. Pregnancy and fetal outcome will be extracted from the clinical record after delivery into specific CRF.

3.10.2 Database

CRF consisted of eligibility form, enrollment form for baseline demographic data, follow-up visit form, PSG form, CPAP adherence form, ultrasound form, delivery form, and neonatal form. Data will be extracted from the CRF into the database. Database will be built with Epidata software to ensure the organization and cross-checking of the data. Data storage will be kept in the computer and hard-disc for back-up at 2 sites (Ramathibodi Sleep Disorder Center and Section of Clinical Epidemiology and Biostatistics: CEB). Additional back-ups of data will be kept in the CEB's server.

3.10.3 Data quality control

Data from each site will be extracted and double entry into the database to ensure correctness of the data within 1 week after data acquisition. Cross-checking and logical check will be done every month by a statistician.

3.10.4 Statistical analysis

- Baseline demographic and clinical variables will be summarized using mean and its standard deviation (SD) or median and inter-quartile range as appropriate for continuous data, whereas categorical variables are summarized in frequencies and percentage. Comparisons of baseline characteristics between the 2 groups will be performed using t-test for the continuous variables and χ^2 test for the categorical variables.

- A blinded evaluation is planned to assess the observed primary end point by a clinical outcome assessment committee.

- The null hypothesis of the primary outcome for the Δ change of blood pressure (DBP) between treatment and controls are tested at 2-sided significant level of p-value <0.05 using t-test.

- Analyses for repeated measurement of secondary outcomes for continuous variables such as 24-hour blood monitoring, glucose level, Uterine artery Doppler ultrasound pulsatile index, estimated fetal growth, at 6-8 weeks after randomization, and during 2-3rd trimesters compared to baseline are performed using a longitudinal data analyses with linear mixed model.

- Analyses of secondary outcome of categorical data such as composite outcome 1 to 3, and individual outcome, control of BP, or non-dipping BP pattern will be compared between CPAP and control groups using χ^2 test**,** or Fisher exact test as appropriate.

- <u>Gestational diabetes, as one of the inclusion criteria, was not included in endpoint analyses as initially planned.</u>

- Predictors for composite outcome, and individual outcomes will be analyzed with multivariate logistic regression model adjusting for other co-variates.

- <u>Statistical analyses will be performed according to the intention-to-treat analysis. Per-protocol analysis will then be analyzed for the comparisons of the results.</u>

- In addition, per-protocol (PPA) and as-treated analyses (ATA) with counterfactual approach were also applied to deal with protocol violation using instrumental variable regression.

- We did not apply imputation for missing data because they were not used for the analysis.

- All statistical analyses are considered significant at the threshold level of 2-sidede p-value < 0.05. Statistical analyses are performed using STATA Version 13.1 (StataCorp, College Station, TX).

- Interim analysis was not performed as initially planned, due to lower than expected number of deliveries when 50% of enrollments were achieved. Decisions were reported to the Ethic committee for approval.
  [51-53].

3.10.5 Termination of subjects

Termination of each subject to continue in the allocated arm will be evoked if one of the below criteria are met:

- Subject's unwillingness during anytime of the study
- Life-threatening condition to the mother such as placenta previa, abruption placenta, severe preeclampsia, eclampsia, HEELP syndrome
- Progression of OSA to severe RDI>30
- Threatening condition to fetus
- Obstetricians concerns

3.10.6 Data Safety Monitoring Board (DSMB)

The concept of clinical equipoise is concerned and adopted in this trial. It is essential and ethical to randomize while uncertainty remains in the community about the merits of the treatment. For the protection of the trial patients, and the future patients whose treatment may be influenced by current trial, data monitoring will be therefore set up [54]. The data safety monitoring committee will take responsibilities as follows:

- Review a protocol and monitor if the protocol is violated
- Recruitment or accrual rate
- Data monitoring and completeness of data
    - Missing data
    - Loss follow up
    - Adverse events
        - Severe rhinitis requiring rescue medication
        - Epistaxis requiring ENT consultation
        - Pneumothorax
        - Excessive air in stomach requiring medication

Interim analysis will be performed 1 time, i.e., when recruitment rate reaches to 50% of total sample size (i.e., ~150) and at the end of the study. Study will be terminated using following stopping rule [54-56]:

- Overall complication (i.e., pneumothorax, severe epistaxis, death, etc.) ≥ 10% in any intervention arm or exceeding the significant level in Pocock interim analysis

- Recruitment rate is 50% lower than as planning during 2 years
- Loss follow-up rate is as high as 30%
- Significant intervention effects using Obrien-Fleming stopping rule as below [51-53]:

**No. of look**	**Interim**	**Level of significance O'Brien-Fleming α_T [111]**
1	Interim 1	0.0052
2	Final	0.048

Work flow for DSMB

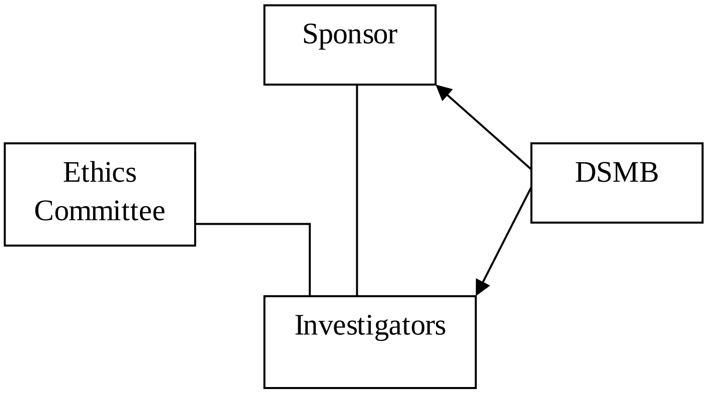

The DSMB will consist of the independent experts not involved in the study enrollment from the essential disciplines including obstetrician, sleep specialist, epidemiologist, and statistician.

Pre-planned interim analysis is intended to monitor the progress and the indications for premature termination of trial [54].

3.10.7 Adverse events

Although, CPAP has been used to treat SDB in pregnancy without any reports of serious side effects, serious and unanticipated adverse events may be anticipated in this population. Data on the following potential adverse events that may be related to the study maneuver will be recorded and evaluated as part of continuous safety monitoring during the trial.

- Severe rhinitis requiring rescue medication
- Epistaxis requiring ENT consultation
- Pneumothorax
- Excessive air in stomach requiring rescue medication

These outcomes will be evaluated on a monthly basis by PI, and if the incidence of any of these outcomes are determined to be 10% greater in any arm of the study, this information will be provided to the PI and committee and the data safety monitoring board (DSMB) for immediate consideration, and evaluated for consideration of termination of the study or treatment arm.

3.10.8 Protocol Violation

Protocol violation includes

- Patients who have been allocated to receive CPAP, but they are later failed to use CPAP following the protocol for any reason.
- Patients who have been allocated to receive a routine ANC but with medical reasons to make them receive CPAP or other intervention/s act like CPAP

- Co-intervention with other intervention such in forms of medication such as anti-hypertensive drug, aspirin, calcium supplement, or other intervention either by physician prescription or non-prescription during the study which might interfere with the result of the study.

Measures to prevent and monitor protocol violation includes

- Close follow up and monitoring of CPAP adherence and early troubleshooting by sleep specialist and sleep technician by means of clinic follow ups, telephone call, and information pamphlet in the CPAP group.
- Close follow up of controls in terms of education regarding general sleep hygiene during ANC follow ups with sleep specialist and information pamphlet.
- Records of medication taken by the subjects will be kept and reviewed for every ANC visit. Education to avoid non-prescription medication during pregnancy will be given.

Protocol violation will be reviewed by the principle investigator who will discuss with the involved staff and provide a written summary including steps taken to avoid future violations.

3.11 Ethical considerations

3.11.1 Informed consent

Pregnant women and/or spouse will be approached during antenatal care for informed consent by researcher or research assistant. Decision to participate or not to participate will not have effect on the subject’s right to standard usual care. In the situation that subject refuses to participate in the study, researcher/co-investigator will ask for the permission to use their demographic data to be included in the analysis if they agree.

3.11.2 The ethics committee approval at all affected sites will be obtained prior to initiation of the study at that site

- The conduct of this research will be performed according to declaration of Helsinki.
- Informed consent must be signed from all subjects in order to participate in the study. Subjects have the right to withdraw from the study at any time, even if she had agreed to participate earlier without interruption of the usual standard care.
- Subject confidentiality will be kept by using non-identifiable subject ID for data collection and analysis.
- In case of any adverse effect occurred during the study that is related to receiving or not receiving intervention, standard care of high-risk pregnancy will be provided to participants.
- Follow-up visits will be according to the usual care for high-risk pregnancy to limit the burden of transportation of the participant.
- Subjects will be provided with incentives for the coverage of transportation for follow up visit.
- Subjects will have the beneficiary of receiving screening for SDB in addition to standard care of high risk pregnancy.
- Ambulatory sleep testing will be performed by certified sleep technician supervised by sleep specialist. Sleep test will be done only when participant is in stable condition to safeguard safety of the subject.
- Discomfort of using CPAP machine will be taken care of by sleep technician and/or sleep specialist by providing troubleshooting as standard care of CPAP usage.

- There will be additional blood sample taken from the subjects not more than 10 ml besides the normal usual care.

3.12 Time frame: Ethics approval valid from 2nd February 2017-27th February 2020

Training& Preparation	October 2016-November 2016
Enrollment& Recruitment	December 2016-October 2019
Data analysis	November 2019- July 2020
Manuscript Preparation	July 2020-

**Months**	**1**	**2**	**3**	**4**	**5**	**6**	**7**	**8**	**9**	**10**	**11**	**12**	**13**	**14**	**15**	**16**	**17**	**18**	**19**	**20**	**21**	**22**	**23**	**24**
**Training& Preparation**	←	→																						
**Enrollment& Recruitment**			←	—	—	—	—	—	—	—	—	—	—	—	—	—	—	—	—	→				
**Data analysis**																						←	→	
**Manuscript Preparation**																							←	→

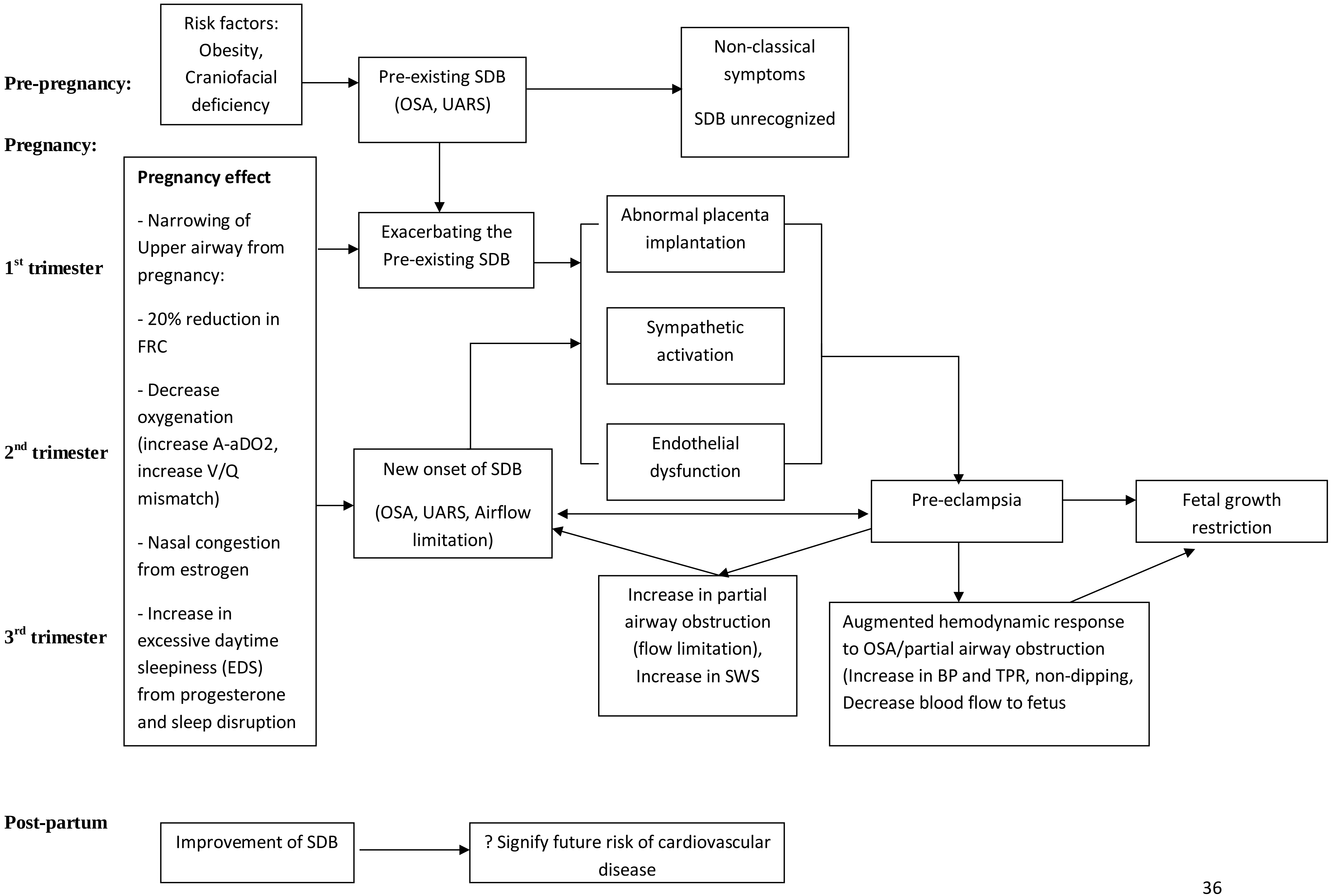
Pre-pregnancy:
Risk factors: Obesity, Craniofacial deficiency
Pre-existing SDB (OSA, UARS)
Non-classical symptoms
SDB unrecognized
Pregnancy:
Pregnancy effect
- Narrowing of Upper airway from pregnancy:
- 20% reduction in FRC
- Decrease oxygenation (increase A-aDO2, increase V/Q mismatch)
- Nasal congestion from estrogen
- Increase in excessive daytime sleepiness (EDS) from progesterone and sleep disruption
1st trimester
2nd trimester
3rd trimester
Exacerbating the Pre-existing SDB
Abnormal placenta implantation
Sympathetic activation
Endothelial dysfunction
New onset of SDB
(OSA, UARS, Airflow limitation)
Pre-eclampsia
Fetal growth restriction
Increase in partial airway obstruction (flow limitation), Increase in SWS
Augmented hemodynamic response to OSA/partial airway obstruction (Increase in BP and TPR, non-dipping, Decrease blood flow to fetus
Post-partum
Improvement of SDB
? Signify future risk of cardiovascular disease

Protocol Flow Chart

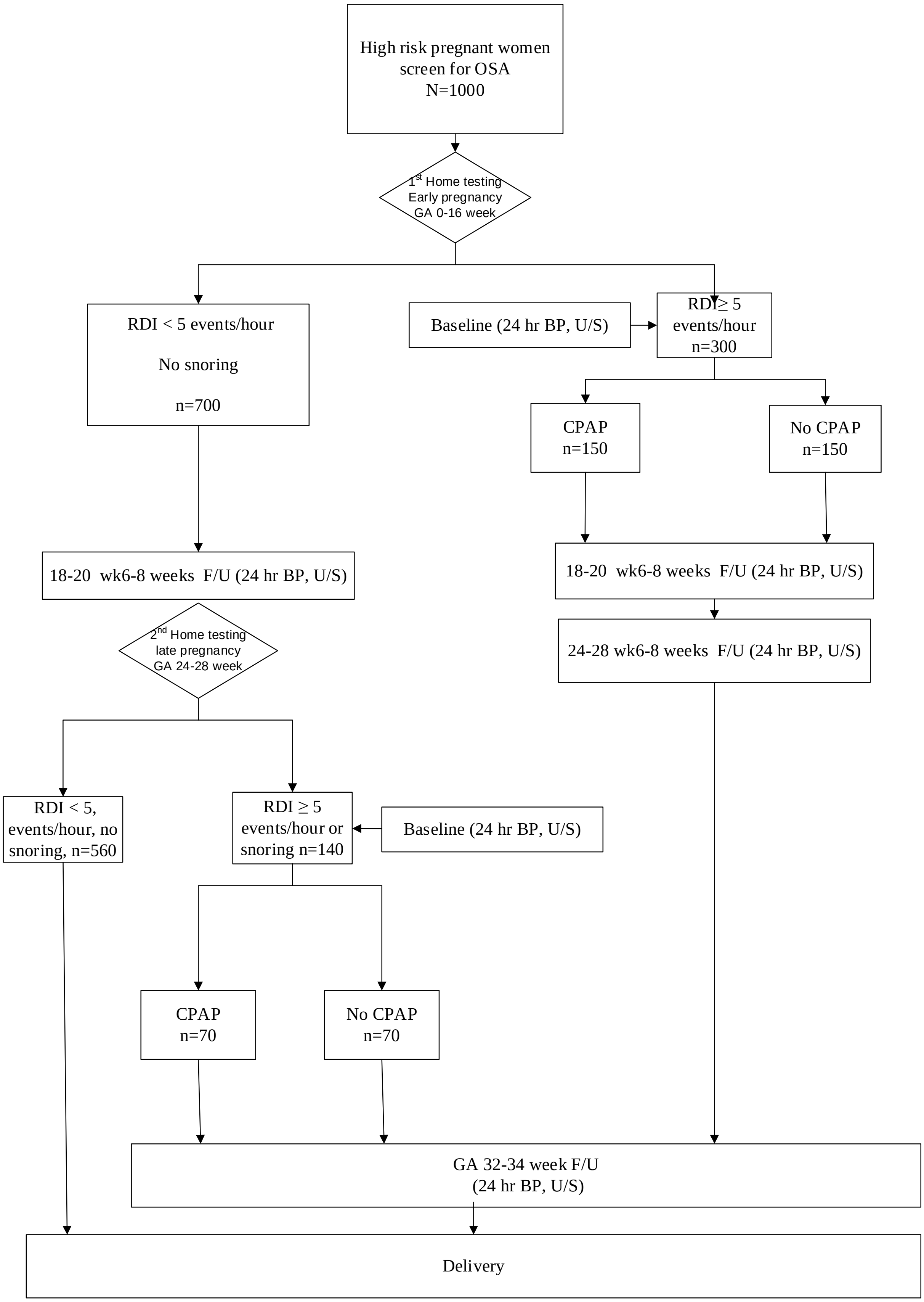

	**Enrollment**	**2nd trimester (6-8 weeks after enrollment)**	**3rd Trimester**	**Delivery**
**Demographic data**	X	X	X	
**Sleep study**	X			
**Systolic blood pressure**	X	X	X	
**Diastolic blood pressure**	X	X	X	
**Target to BP control**	X	X	X	
**24-hr ambulatory BP**	X	X	X	
**PSQI Questionnaire**	X	X	X	
**ESS**	X	X	X	
**Fetal ultrasound**	X	X	X	
**PIH/preeclampsia event**		X	X	X
**Obstetric complication**				X
**Infant outcome**				X
**Placenta character**				X
**CPAP adherence (only CPAP group)**		X	X	X

REFERENCES

1. Somers VK, Dyken ME, Clary MP, Abboud FM. Sympathetic neural mechanisms in obstructive sleep apnea. The Journal of clinical investigation. 1995;96(4):1897-904.

2. Young T, Palta M, Dempsey J, Skatrud J, Weber S, Badr S. The occurrence of sleep-disordered breathing among middle-aged adults. N Engl J Med. 1993;328(17):1230-5.

3. Shahar E, Whitney CW, Redline S, Lee ET, Newman AB, Nieto FJ, et al. Sleep-disordered breathing and cardiovascular disease: cross-sectional results of the Sleep Heart Health Study. Am J Respir Crit Care Med. 2001;163(1):19-25.

4. Suwanprathes P, Won C, Komoltri C, Nana A, Kotchabhakdi N, Guilleminault C. Epidemiology of sleep-related complaints associated with sleep-disordered breathing in Bangkok, Thailand. Sleep Med. 2010;11(10):1025-30.

5. Neruntarat C, Chantapant S. Prevalence of sleep apnea in HRH Princess Maha Chakri Srinthorn Medical Center, Thailand. Sleep & breathing = Schlaf & Atmung. 2011;15(4):641-8.

6. Young T, Evans L, Finn L, Palta M. Estimation of the clinically diagnosed proportion of sleep apnea syndrome in middle-aged men and women. Sleep. 1997;20(9):705-6.

7. Fung AM, Wilson DL, Barnes M, Walker SP. Obstructive sleep apnea and pregnancy: The effect on perinatal outcomes. Journal of Perinatology. 2012;32(6):399-406.

8. Olivarez SA, Ferres M, Antony K, Mattewal A, Maheshwari B, Sangi-Haghpeykar H, et al. Obstructive sleep apnea screening in pregnancy, perinatal outcomes, and impact of maternal obesity. American Journal of Perinatology. 2011;28(8):651-8.

9. Tantrakul V, Sirijanchune P, Panburana P, Pengjam J, Suwansathit W, Boonsarngsuk V, et al. Screening of Obstructive Sleep Apnea during Pregnancy: Differences in Predictive Values of Questionnaires across Trimesters. Journal of clinical sleep medicine : JCSM : official publication of the American Academy of Sleep Medicine. 2015;11(2):157-63.

10. Wilson DL, Walker SP, Fung AM, O'Donoghue F, Barnes M, Howard M. Can we predict sleep-disordered breathing in pregnancy? The clinical utility of symptoms. Journal of Sleep Research. 2013;22(6):670-8.

11. Facco FL, Ouyang DW, Zee PC, Grobman WA. Development of a pregnancy-specific screening tool for sleep apnea. Journal of Clinical Sleep Medicine. 2012;8(4):389-94.

12. Antony KM, Agrawal A, Arndt ME, Murphy AM, Alapat PM, Guntupalli KK, et al. Obstructive sleep apnea in pregnancy: Reliability of prevalence and prediction estimates. Journal of Perinatology. 2014;34(8):587-93.

13. Tantrakul V, Numthavaj P., Guilleminault C., McEvoy M., Panburana P., Khaing W., Attia J., Thakkinstian A. Performance of screening questionnaires for obstructive sleep apnea during pregnancy: A systematic review and meta-analysis. 2016.

14. Pamidi S, Pinto LM, Marc I, Benedetti A, Schwartzman K, Kimoff RJ. Maternal sleep-disordered breathing and adverse pregnancy outcomes: A systematic review and metaanalysis. American Journal of Obstetrics and Gynecology. 2014;210(1):52.e1-.e14.

15. Xu T, Feng Y, Peng H, Guo D, Li T. Obstructive Sleep Apnea and the Risk of Perinatal Outcomes: A Meta-Analysis of Cohort Studies. Scientific Reports. 2014;4.

16. Ding XX, Wu YL, Xu SJ, Zhang SF, Jia XM, Zhu RP, et al. A systematic review and quantitative assessment of sleep-disordered breathing during pregnancy and perinatal outcomes. Sleep and Breathing. 2014:1-11.

17. Hedman C, Pohjasvaara T, Tolonen U, Suhonen-Malm AS, Myllyla VV. Effects of pregnancy on mothers' sleep. Sleep Med. 2002;3(1):37-42.

18. Berry RB, Budhiraja R, Gottlieb DJ, Gozal D, Iber C, Kapur VK, et al. Rules for scoring respiratory events in sleep: update of the 2007 AASM Manual for the Scoring of Sleep and Associated Events. Deliberations of the Sleep Apnea Definitions Task Force of the

American Academy of Sleep Medicine. Journal of clinical sleep medicine : JCSM : official publication of the American Academy of Sleep Medicine. 2012;8(5):597-619.

19. Kushida CA, Littner MR, Morgenthaler T, Alessi CA, Bailey D, Coleman J, Jr., et al. Practice parameters for the indications for polysomnography and related procedures: an update for 2005. Sleep. 2005;28(4):499-521.

20. Guilleminault C, Palombini L, Poyares D, Takaoka S, Huynh NT, El-Sayed Y. Pre-eclampsia and nasal CPAP: part 1. Early intervention with nasal CPAP in pregnant women with risk-factors for pre-eclampsia: preliminary findings. Sleep Med. 2007;9(1):9-14.

21. Poyares D, Guilleminault C, Hachul H, Fujita L, Takaoka S, Tufik S, et al. Pre-eclampsia and nasal CPAP: part 2. Hypertension during pregnancy, chronic snoring, and early nasal CPAP intervention. Sleep Med. 2007;9(1):15-21.

22. Epstein LJ, Kristo D, Strollo PJ, Jr., Friedman N, Malhotra A, Patil SP, et al. Clinical guideline for the evaluation, management and long-term care of obstructive sleep apnea in adults. Journal of clinical sleep medicine : JCSM : official publication of the American Academy of Sleep Medicine. 2009;5(3):263-76.

23. Marin JM, Carrizo SJ, Vicente E, Agusti AG. Long-term cardiovascular outcomes in men with obstructive sleep apnoea-hypopnoea with or without treatment with continuous positive airway pressure: an observational study. Lancet. 2005;365(9464):1046-53.

24. Kendzerska T, Gershon AS, Hawker G, Leung RS, Tomlinson G. Obstructive Sleep Apnea and Risk of Cardiovascular Events and All-Cause Mortality: A Decade-Long Historical Cohort Study. PLoS Medicine. 2014;11(2).

25. Fava C, Dorigoni S, Dalle Vedove F, Danese E, Montagnana M, Guidi GC, et al. Effect of CPAP on blood pressure in patients with OSA/hypopnea a systematic review and meta-analysis. Chest. 2014;145(4):762-71.

26. Jerath R, Barnes VA, Fadel HE. Mechanism of development of pre-eclampsia linking breathing disorders to endothelial dysfunction. Medical Hypotheses. 2009;73(2):163-6.
27. Guilleminault C, Kreutzer M, Chang JL. Pregnancy, sleep disordered breathing and treatment with nasal continuous positive airway pressure. Sleep Medicine. 2004;5(1):43-51.
28. Sateia M. International Classification of Sleep Disorders. 3rd edition ed: Darien, IL: American Academy of Sleep Medicine; 2014.
29. Hypertension in pregnancy. Report of the American College of Obstetricians and Gynecologists' Task Force on Hypertension in Pregnancy. Obstet Gynecol. 2013;122(5):1122-31.
30. Classification and diagnosis of diabetes. Diabetes Care. 2015;38 Suppl:S8-s16.
31. Chabra S. Consistent definition of preterm birth: a research imperative! Am J Obstet Gynecol. 2016;214(4):552.
32. Chabra S. Concept of gestational age in "completed Weeks": Lost in translation. Obstetrics and Gynecology. 2012;119(1):183-4.
33. Palombini LO, Tufik S, Rapoport DM, Ayappa IA, Guilleminault C, de Godoy LB, et al. Inspiratory flow limitation in a normal population of adults in Sao Paulo, Brazil. Sleep. 2013;36(11):1663-8.
34. Parati G, Stergiou G, O'Brien E, Asmar R, Beilin L, Bilo G, et al. European Society of Hypertension practice guidelines for ambulatory blood pressure monitoring. J Hypertens. 2014;32(7):1359-66.
35. O'Brien E, Parati G, Stergiou G, Asmar R, Beilin L, Bilo G, et al. European Society of Hypertension position paper on ambulatory blood pressure monitoring. J Hypertens. 2013;31(9):1731-68.

36. Hermida RC, Ayala DE, Fontao MJ, Mojon A, Fernandez JR. Ambulatory blood pressure monitoring: importance of sampling rate and duration--48 versus 24 hours--on the accurate assessment of cardiovascular risk. Chronobiology international. 2013;30(1-2):55-67.

37. Brown MA. Is there a role for ambulatory blood pressure monitoring in pregnancy? Clinical and experimental pharmacology & physiology. 2014;41(1):16-21.

38. Gupta HP, Singh RK, Singh U, Mehrotra S, Verma NS, Baranwal N. Circadian pattern of blood pressure in normal pregnancy and preeclampsia. Journal of obstetrics and gynaecology of India. 2011;61(4):413-7.

39. Bhide A, Sankaran S, Moore J, Khalil A, Furneaux E. Ambulatory blood pressure measurements in mid-pregnancy and development of hypertensive pregnancy disorders. Hypertension in pregnancy. 2014;33(2):159-67.

40. Ayala DE, Hermida RC. Ambulatory blood pressure monitoring for the early identification of hypertension in pregnancy. Chronobiology international. 2013;30(1-2):233-59.

41. Eguchi K, Ohmaru T, Ohkuchi A, Hirashima C, Takahashi K, Suzuki H, et al. Ambulatory BP monitoring and clinic BP in predicting small-for-gestational-age infants during pregnancy. Journal of human hypertension. 2016;30(1):62-7.

42. Bilo G, Zorzi C, Ochoa Munera JE, Torlasco C, Giuli V, Parati G. Validation of the Somnotouch-NIBP noninvasive continuous blood pressure monitor according to the European Society of Hypertension International Protocol revision 2010. Blood pressure monitoring. 2015;20(5):291-4.

43. Bhide A, Acharya G, Bilardo CM, Brezinka C, Cafici D, Hernandez-Andrade E, et al. ISUOG practice guidelines: Use of Doppler ultrasonography in obstetrics. Ultrasound in Obstetrics and Gynecology. 2013;41(2):233-9.

44. Cnossen JS, Morris RK, Ter Riet G, Mol BWJ, Van Der Post JAM, Coomarasamy A, et al. Use of uterine artery Doppler ultrasonography to predict pre-eclampsia and intrauterine growth restriction: A systematic review and bivariable meta-analysis. CMAJ. 2008;178(6):701-11.

45. Diguisto C, Piver E, Le Gouge A, Eboue F, Le Vaillant C, Marechaud M, et al. First trimester uterine artery Doppler, sFlt-1 and PlGF to predict preeclampsia in a high-risk population. The journal of maternal-fetal & neonatal medicine : the official journal of the European Association of Perinatal Medicine, the Federation of Asia and Oceania Perinatal Societies, the International Society of Perinatal Obstet. 2016:1-17.

46. Gunenc O, Cicek N, Gorkemli H, Celik C, Acar A, Akyurek C. The effect of methyldopa treatment on uterine, umblical and fetal middle cerebral artery blood flows in preeclamptic patients. Arch Gynecol Obstet. 2002;266(3):141-4.

47. Khalil A, Harrington K, Muttukrishna S, Jauniaux E. Effect of antihypertensive therapy with alpha-methyldopa on uterine artery Doppler in pregnancies with hypertensive disorders. Ultrasound in obstetrics & gynecology : the official journal of the International Society of Ultrasound in Obstetrics and Gynecology. 2010;35(6):688-94.

48. Folic MM, Jankovic SM, Varjacic MR, Folic MD. Effects of methyldopa and nifedipine on uteroplacental and fetal hemodynamics in gestational hypertension. Hypertension in pregnancy. 2012;31(1):31-9.

49. Lowe LP, Metzger BE, Dyer AR, Lowe J, McCance DR, Lappin TR, et al. Hyperglycemia and Adverse Pregnancy Outcome (HAPO) Study: associations of maternal A1C and glucose with pregnancy outcomes. Diabetes Care. 2012;35(3):574-80.

50. Reid J, Taylor-Gjevre R, Gjevre J, Skomro R, Fenton M, Olatunbosun F, et al. Can gestational hypertension be modified by treating nocturnal airflow limitation? Journal of Clinical Sleep Medicine. 2013;9(4):311-7.

51. DeMets DL, Lan KK. Interim analysis: the alpha spending function approach. Statistics in medicine. 1994;13(13-14):1341-52; discussion 53-6.

52. Falissard B, Lellouch J. The succession procedure for interim analysis: extensions for early acceptance of Ho and for flexible times of analysis. Statistics in medicine. 1993;12(1):51-67.

53. Pocock SJ. Current controversies in data monitoring for clinical trials. Clinical trials (London, England). 2006;3(6):513-21.

54. S P. Clinical Trials. A methodologic perspective: John Wiley & Sons, Inc., Hoboken, New Jersey; 2005.

55. Lachin JM. A review of methods for futility stopping based on conditional power. Statistics in medicine. 2005;24(18):2747-64.

56. Lachin JM. Futility interim monitoring with control of type I and II error probabilities using the interim Z-value or confidence limit. Clinical trials (London, England). 2009;6(6):565-73.
