# Supplementary material for: Treatment of obstructive sleep apnea in high risk pregnancy: a multicenter randomized controlled trial
Source: Respir Res. 2023 Jun 27;24:171. doi: 10.1186/s12931-023-02445-y (PMC10294320; doi:10.1186/s12931-023-02445-y)
Supplement: Supplementary file 2 — Additional file 2. Additional. [file 12931_2023_2445_MOESM2_ESM.docx]

**Additional Online Content**

Tantrakul V, Ingsathit A, Liamsombut S, et al; Treatment of obstructive sleep apnea in high risk pregnancy: A multicenter randomized controlled trial.

**Page**

**List of Ethical Committee Review Board for all sites** 3

**Figure S1** The temporal changes of **systolic blood pressure** during pregnancy in

**hypertensive participants** **taking anti-hypertensive medication** in CPAP

versus usual-care (no CPAP) groups using the modified intention-to-treat

and per-protocol analyses. 4

**Figure S2** The temporal changes of **diastolic blood pressure** during pregnancy in

**hypertensive participants taking anti-hypertensive medication** in CPAP

versus usual-care (no CPAP) groups using the modified intention-to-treat

and per-protocol analyses. 5

**Figure S3** The temporal changes of **systolic blood pressure** during pregnancy in

**participants NOT-taking anti-hypertensive medication** in CPAP versus

usual-care (no CPAP) groups using the modified intention-to-treat and

per-protocol analyses. 6

**Figure S4** The temporal changes of **diastolic blood pressure** during pregnancy in

**participants NOT-taking anti-hypertensive medication** in CPAP versus

usual-care (no CPAP) groups using the modified intention-to-treat and

per-protocol analyses. 7

**Table S1** Missing data 8

**Table S2** Baseline characteristics of CPAP-adherent(average-CPAP use≥4 hour/night)

and non-adherent (average-CPAP use<4 hour/night) users in participants

randomized to CPAP intervention 9

**Table S3** Details on CPAP usage on the study participants randomized to the

intervention group 11

**Table S4** Comparison of other secondary outcomes between intervention

groups on the modifiend intention-to-treat analysis 13

**Table S5** Adverse events by intervention groups**.**  15

**Table S6** Primary outcomes on blood pressures using per-protocol and counterfactual

analyses 16

**Table S7** Post-hoc analyses according to participants with mild OSA/UARS and OSA

subgroups on primary outcomes of blood pressures using modified intention-to-treat

analysis 18

**Table S8** Post-hoc analyses according to participants with mild OSA/UARS and OSA

subgroups on secondary outcomes of preeclampsia and gestational hypertensive

complications using modified intention-to-treat analysis 19

**Table S9** A sensitivity analysis excluding participants with new-onset OSA randomized

during 2^nd^ trimester on primary outcomes of blood pressures using modified

intention-to-treat analysis 21

**Table S10** A sensitivity analysis excluding participants with new-onset OSA randomized

during 2^nd^ trimester on secondary outcomes of preeclampsia and gestational

hypertensive complications using modified intention-to-treat analysis 22

**List of Ethical Committee Review Board for all sites and study numbers**

1. **Committee on Human Rights Related to Research Involving Human Subjects, Faculty of Mediciane Ramathibodi Hospital, Mahidol University.**

**Study No.** ID 11-58-12

1. **Ethics Committee on Researches involving Human, Rajavithi Hospital**

**Study No.** 60117

1. **Instutional Review Board, Royal Thai Army Medical Department (for Phramongkutklao Hospital)**

**Study No.** Q031h/60


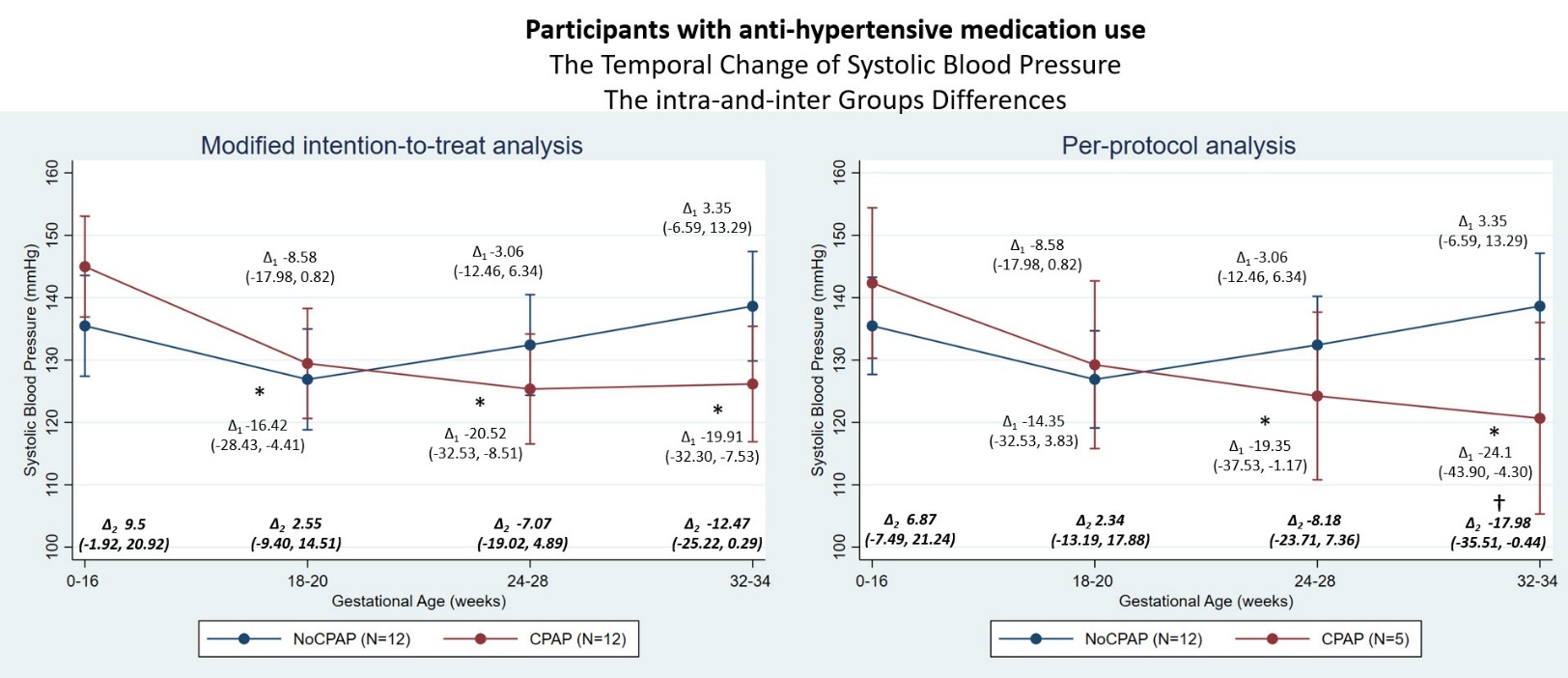


**Figure S1.** The temporal changes of **systolic blood pressure** during pregnancy in **hypertensive participants taking anti-hypertensive medication** in CPAP versus usual-care (no CPAP) groups using the modified intention-to-treat and per-protocol analyses.

**Note:** The BP nadir-point and the increase thereafter occurred earlier at 18-20 weeks in those taking anti-hypertensive in the usual-care group. But this earlier nadir trend did not occur in the CPAP group

**Intra-group changes: ∆_1_ =difference within groups compared to baseline (gestational age <16 weeks); * Denotes *p*-value<0.05**

CPAP group (lower panel); usual-care group (upper panel)

**Inter-group changes: ∆_2_ =difference between groups during each timepoints; †Denotes *p*-value<0.05**


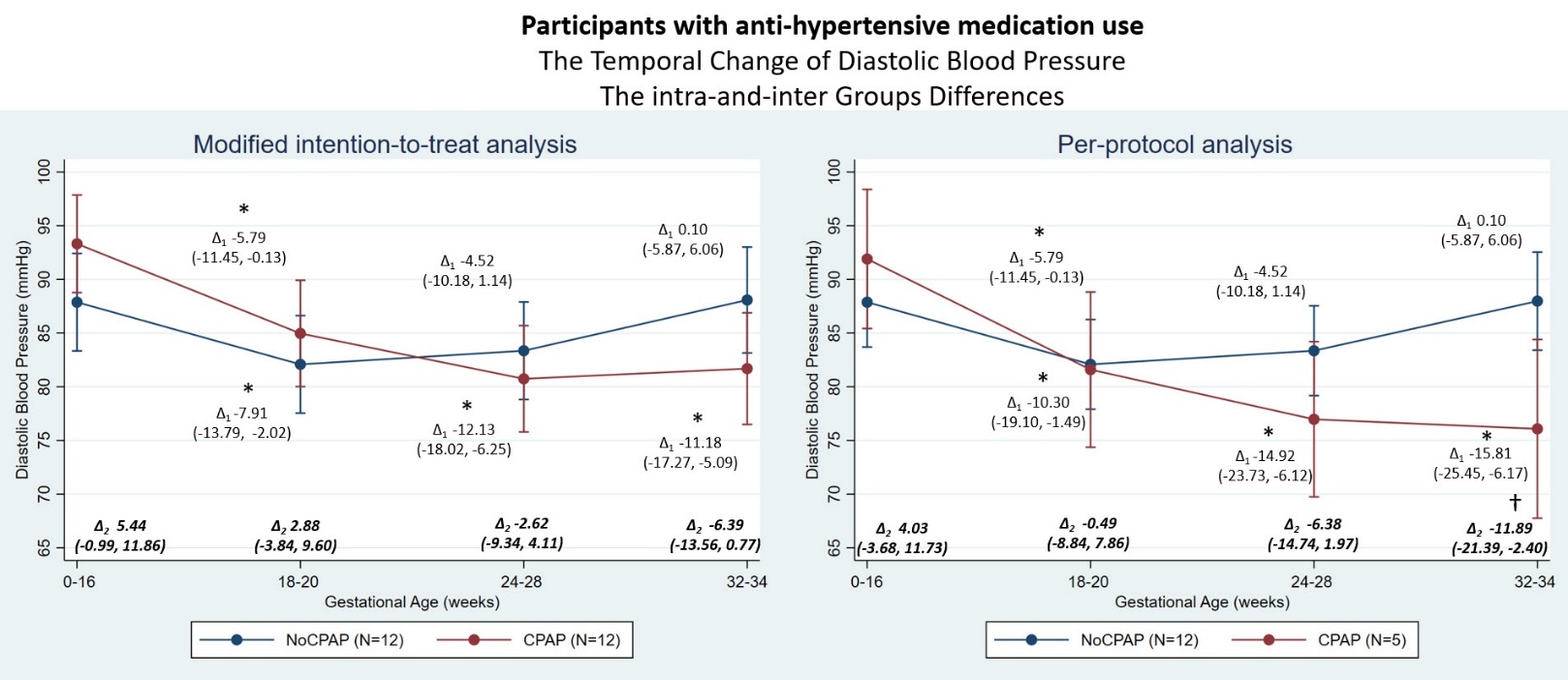


**Figure S2.** The temporal changes of **diastolic blood pressure** during pregnancy in **hypertensive participants taking anti-hypertensive medication** in CPAP versus usual-care (no CPAP) groups using the modified intention-to-treat and per-protocol analyses.

**Note:** The BP nadir-point and the increase thereafter occurred earlier at 18-20 weeks in those taking anti-hypertensive in the usual-care group. But this earlier nadir trend did not occur in the CPAP group.

**Intra-group changes: ∆_1_ =difference within groups compared to baseline (gestational age <16 weeks); * Denotes *p*-value<0.05**

CPAP group (lower panel); usual-care group (upper panel)

**Inter-group changes: ∆_2_ =difference between groups during each timepoints; †Denotes *p*-value<0.05**


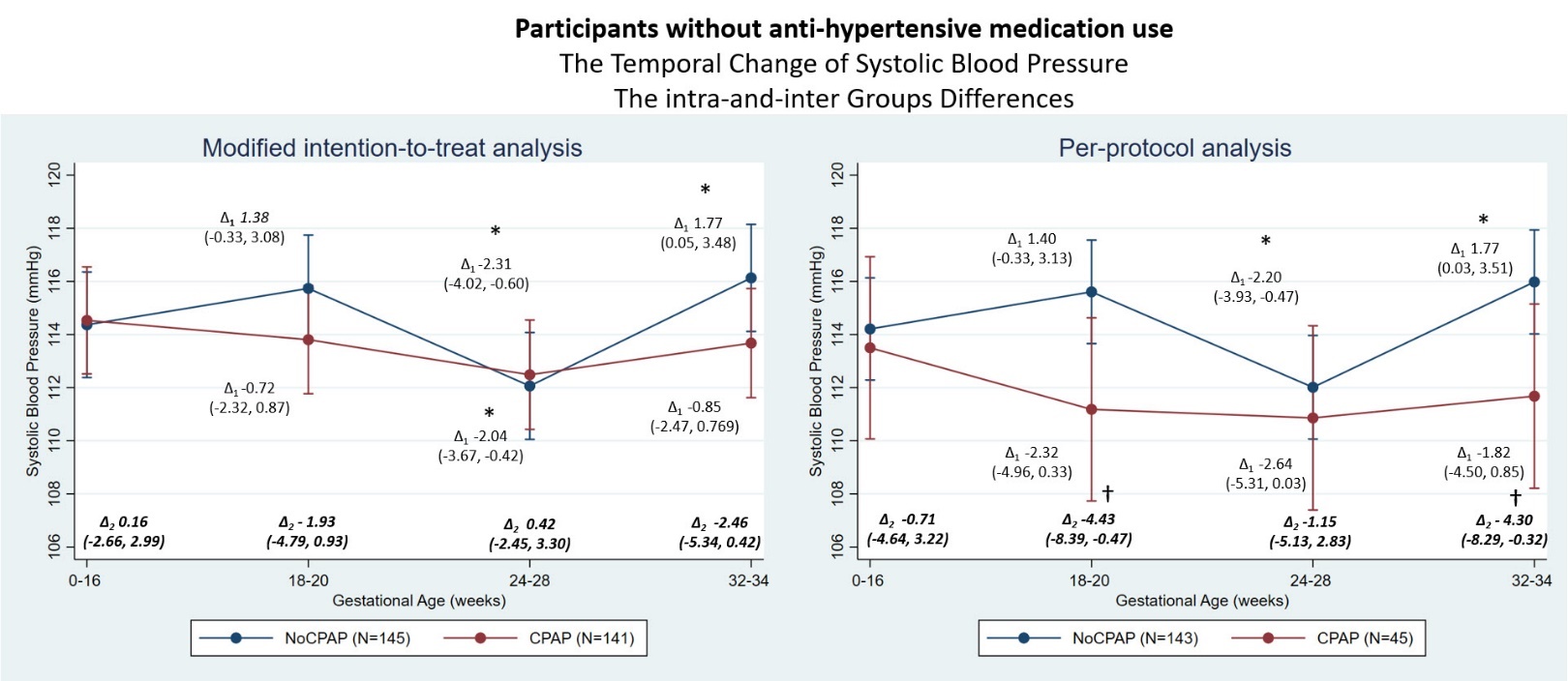


**Figure S3.** The temporal changes of **systolic blood pressure** during pregnancy in **participants NOT-taking anti-hypertensive medication** in CPAP versus usual-care (no CPAP) groups using the modified intention-to-treat and per-protocol analyses.

**Note:** The BP nadir-point occurred at 24-28 weeks in both CPAP and usual-care groups.

**Intra-group changes: ∆_1_ =difference within groups compared to baseline (gestational age <16 weeks); * Denotes *p*-value<0.05**

CPAP group (lower panel); usual-care group (upper panel)

**Inter-group changes: ∆_2_ =difference between groups during each timepoints; †Denotes *p*-value<0.05**


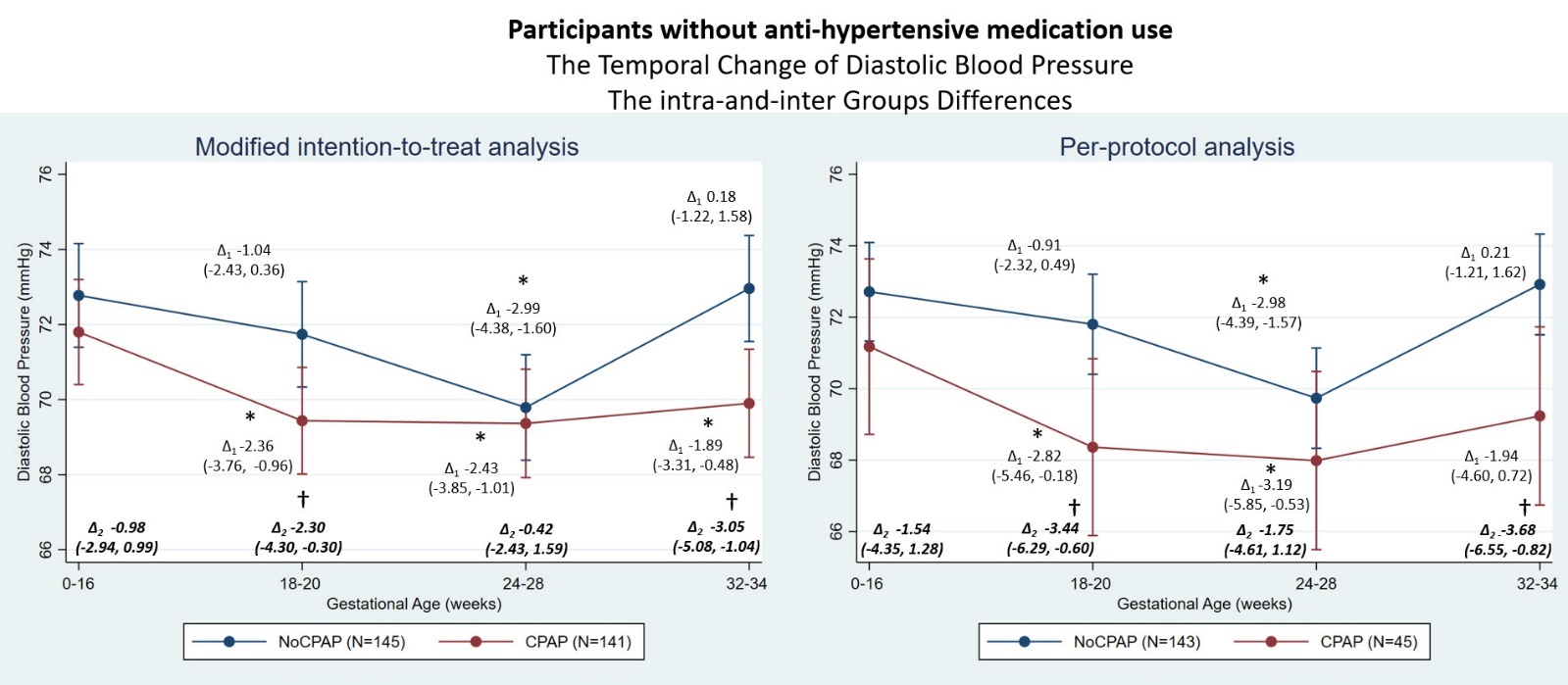


**Figure S4.** The temporal changes of **diastolic blood pressure** during pregnancy in **participants NOT-taking anti-hypertensive medication** in CPAP versus usual-care (no CPAP) groups using the modified intention-to-treat and per-protocol analyses.

**Note:** The BP nadir-point occurred at 24-28 weeks in both CPAP and usual-care groups.

**Intra-group changes: ∆_1_ =difference within groups compared to baseline (gestational age <16 weeks); * Denotes *p*-value<0.05**

CPAP group (lower panel); usual-care group (upper panel)

**Inter-group changes: ∆_2_ =difference between groups during each timepoints; †Denotes *p*-value<0.05**

**Table S1. Missing data**

| **Factors^a^** | **Total** | **Number of Missing** | **Percent of Missing** |
| --- | --- | --- | --- |
| Age | 310 | 0 | 0 |
| Gestational Age | 310 | 0 | 0 |
| Pre-pregnancy body mass index | 310 | 0 | 0 |
| Body mass index at enrollment | 310 | 0 | 0 |
| Neck circumference | 310 | 0 | 0 |
| Waist circumference | 310 | 1 | 0.32 |
| Nulliparous | 310 | 0 | 0 |
| Snoring reports | 310 | 0 | 0 |
| Systolic blood pressure | 310 | 0 | 0 |
| Diastolic blood pressure | 310 | 0 | 0 |
| Mean blood pressure | 310 | 0 | 0 |
| Fasting plasma glucose | 310 | 20 | 6.45 |
| Hemoglobin A1C | 310 | 116 | 37.42 |
| Respiratory disturbance index (RDI) | 310 | 0 | 0 |
| ESS | 310 | 2 | 0.65 |
| Preeclampsia | 310 | 0 | 0 |
| Preterm birth | 310 | 0 | 0 |
| Cesarean section rate | 310 | 0 | 0 |
| Body weight at delivery | 310 | 1 | 0.32 |
| Weight gain at delivery | 310 | 0 | 0 |
| Infant birth weight, gram | 310 | 0 | 0 |
| Low-infant birth weight | 310 | 0 | 0 |
| APGAR 1 min score | 310 | 43 | 13.87 |
| APGAR 5 min score | 310 | 10 | 3.23 |
| NICU admission | 310 | 0 | 0 |
| Neonatal admission day | 310 | 1 | 0.32 |
| Maternal admission day | 310 | 0 | 0 |
| Composite 1 | 310 | 0 | 0 |
| Composite 2 | 310 | 0 | 0 |
| Composite 3 | 310 | 0 | 0 |
| Gestational hypertension | 310 | 0 | 0 |
| Severe preeclampsia | 310 | 0 | 0 |
| Gestational age at delivery | 310 | 0 | 0 |
| Fetal growth restriction | 310 | 0 | 0 |
| Small for gestational age (term+birthweight<2500) | 310 | 0 | 0 |
| Body weight child >=4000 gram | 310 | 0 | 0 |

Missing data rates were 0% to 37.2%, in which highest in hemoglobin A1C because it was not a routine lab test. Given that the primary outcome of blood pressures and important secondary outcomes (preeclampsia, pregnancy-induced hypertension) were not missing, data imputation was not necessarily applied.

**Table S2.** **Baseline characteristics of CPAP-adherent**(average-CPAP use≥4 hour/night)  **and non-adherent** (average-CPAP use<4 hour/night) **users in participants randomized to CPAP intervention**

| **Baseline characteristics** | **Adherent CPAP users**  **n=50 (32.7%)** | **Non-adherent CPAP users**  **N=103 (67.3%)** | ***p*-value** |
| --- | --- | --- | --- |
| Age (year), mean (SD) | 33.53±5.15 | 32.65 ±5.33 | 0.333 |
| Gestational age at randomization (week), mean (SD) | 13.6±3.6 | 15.9±5.1 | 0.004 |
| Nulliparous, no. (%) | 17 (34.0) | 32 (31.1) | 0.715 |
| Chronic hypertension, no. (%) | 11 (22.0) | 16 (15.5) | 0.325 |
| Pre-pregnancy obesity, no. (%) | 29 (58.0) | 70 (68.0) | 0.227 |
| Gestational diabetes detected early, no. (%) | 24 (48.0) | 48 (46.6) | 0.871 |
| Anti-hypertensive medication, no. (%) | 5 (10.0) | 7 (6.8) | 0.489 |
| Anthropometric measurement |  |  |  |
| Pre-pregnancy body mass index (kg/m^2^), mean (SD) | 28.32±6.73 | 29.57±5.33 | 0.21 |
| Body mass index at enrollment (kg/m^2^), mean (SD) | 29.26±6.44 | 30.56±5.68 | 0.20 |
| Neck circumference (cm),  mean (SD) | 35.33±4.37 | 35.41±2.57 | 0.89 |
| Waist circumference (cm),  mean (SD) | 95.37±13.56 | 98.83±12.0 | 0.11 |
| Obstructive sleep apnea characteristics |  |  |  |
| Respiratory disturbance index ^a^ (events/hour), mean (SD) | 14.54±6.7 | 14.13±6.7 | 0.72 |
| Apnea-hypopnea index ^b^ (events/hour), median (IQR) | 7.25(4.55, 13.15) | 6.80(4.30, 12.40) | 0.557 |
| Oxygen desaturation index^c^ (events/hour), median (IQR) | 1.75(0.30, 4.10) | 2.0(0.80, 5.00) | 1.00 |
| Average oxygen saturation (%), mean (SD) | 96.94±0.99 | 96.63±1.08 | 0.09 |
| Minimum oxygen saturation (%), mean (SD) | 90.18±4.18 | 89.06±4.12 | 0.120 |
| Report of frequent snoring ≥3 times/week^d^, no. (%) | 24(49.0) | 51(49.5) | 0.951 |
| Epworth Sleepiness Scale, median (IQR) | 7.88(5, 10) | 8.8(6, 12) | 0.16 |
| Systolic blood pressure (mmHg), mean (SD) | 116.39±15.46 | 117.18±16.48 | 0.78 |
| Diastolic blood pressure (mmHg), mean (SD) | 73.25±11.73 | 73.60±11.11 | 0.86 |
| Mean blood pressure (mmHg),  mean (SD) | 87.63±12.28 | 88.13±12.55 | 0.82 |
| Fasting plasma glucose (mg/dL),  mean (SD) | 99.78 ±33.35 | 98.04±23.08 | 0.70 |
| Hemoglobin A1C (mg/dL), mean (SD) | 5.44±0.98 | 5.42±0.72 | 0.91 |
| Site of randomization, no. (%) |  |  | 0.27 |
| Ramathibodi Hospital | 36(72.0) | 64(62.1) |  |
| Rajvithi Hospital | 5(10.0) | 21(20.4) |  |
| Pramongkutklao Hospital | 9(18.0) | 18(17.5) |  |
| Education level, Bachelor^d^, no. (%) | 20(40.0) | 43(41.8) | 0.75 |

**Abbreviations:** CPAP, continuous positive airway pressure; IQR, interquartile range; SD, standard deviation

^a^ The respiratory disturbance index (RDI) is the number of apneas, hypopneas and respiratory-event related arousal events per hour of sleep. ^b^The apnea-hypopnea index is the number of apneas, and hypopneas events per hour of sleep. ^c^The oxygen desaturation index is the number of times per hour of sleep during the oximeter recording that the oxygen saturation drops by at least 3 percentage points from baseline. ^d^Snoring and education level were self-reported by the participants based on questionnaire in case record form

**Table S3. Details on CPAP usage on the study participants randomized to the intervention group**

|  | **Total CPAP Group**  **N=153** | **Adherent CPAP users***  **N=50**  **(32.7%)** | **Non-adherent CPAP users***  **N=103 (67.3%)** | ***p-*value** |
| --- | --- | --- | --- | --- |
| **CPAP usage data**^a^ |  |  |  |  |
| Average nightly CPAP use (hour) |  |  |  |  |
| Mean (SD)  Median (IQR) | 2.5±2.5  1.7 (0.2, 4.5) | 5.6±.2  5.4 (4.6, 6.4) | 1.0±1.1  0.4 (0.1, 1.9) |  |
| Auto-adjusting CPAP |  |  |  |  |
| Minimum pressure (cmH_2_O),  mean (SD)  Maximum pressure (cmH_2_O), mean (SD) | 4.9±1.3  8.2±1.8 | 5.2±1.2  8.2±1.6 | 4.7±1.3  8.2±1.9 | 0.082 |
| 90 percentile pressure (cmH_2_O),  mean (SD) | 6.3±1.3 | 6.4±1.2 | 6.3±1.2 | 0.54 |
| Apnea-hypopnea index (events/hour), median (IQR) | 0.8(0.4, 1.3) | 0.7(0.4, 1.2) | 0.9(0.4, 1.4) | 0.22 |
| Central apnea index (events/hour), median (IQR) | 0.1(0.0, 0.1) | 0(0,0) | 0.1(0, 0.2) | 0.81 |
| Obstructive apnea index (events/hour), median (IQR) | 0.1(0, 0.1) | 0.1(0, 0.1) | 0.1(0, 0.2) | 0.24 |
| Hypopnea index (events/hour), median (IQR) | 0.6(0.3, 1.1) | 0.5(0.4, 1.1) | 0.7(0.3, 1.1) | 0.37 |
| Flow limitation index (events/hour), median (IQR) | 0.8 (0.4, 1.3) | 0.6 (0.5, 1.3) | 0.9 (0.4, 1.2) | 0.366 |
| Periodic breathing (%), median (IQR) | 0.1 (0, 0.2) | 0.2(0.2, 0.2) | 0(0,0) | 0.776 |
| Large leak (%), median (IQR) | 0.2(0.0, 0.5) | 0.2(0, 0.4) | 0.1(0, 3.7) | 0.361 |
| **CPAP side effects ^b^,** no. (%) |  |  |  |  |
| Rhinitis, no. (%) |  |  |  | 0.303 |
| No symptoms | 78(50.9) | 26(52.0) | 52(50.5) |  |
| Mild symptoms | 48(31.4) | 17(34.0) | 31(30.1) |  |
| Symptoms requiring medication | 27(17.7) | 7(14.0) | 20(19.4) |  |
| Epistaxis, no. (%) |  |  |  |  |
| No symptoms | 152(99.4) | 50(100) | 102(99.0) | 0.409 |
| Mild symptoms | 1(0.6) | 0 | 1(1.0) |  |
| Symptoms requiring treatment | 0(0) | 0 | 0 |  |
| Mask leakage, no. (%) | 22(14.4) | 10(20.0) | 12(11.7) | 0.167 |
| Mask pressure sore, no. (%) | 19(12.4) | 8(16.0) | 11(10.7) | 0.349 |
| Pressure intolerance, no. (%) | 43(28.1) | 11(22.0) | 32(31.1) | 0.242 |
| Mouth breathing, no. (%) | 6(3.9) | 2(4.0) | 4(3.9) | 0.972 |
| Bloating, no. (%) |  |  |  |  |
| No symptoms | 140(92.1) | 47(94.0) | 93(91.2) | 0.713 |
| Mild symptoms | 11(7.2) | 3(6.0) | 8(7.8) |  |
| Symptoms requiring treatment | 1(0.7) | 0(0) | 1(1.0) |  |

**Abbreviations:** CPAP, continuous positive airway pressure; IQR, interquartile range; SD, standard deviation

***** Adherence defined as average-CPAP use ≥4 hours/night; non-adherence defined as average-CPAP use < 4 hours/night.

^a^ Usage data were recorded within the memory card and downloaded including duration from randomization to delivery

^b^ CPAP side effects were based on the participants’ self-reports and questionnaire and recorded in the case record form

**Table S4. Comparison of other secondary outcomes between intervention groups using the modified intention-to-treat analysis**

| **Endpoints** | **CPAP**  **Group**  **(n=153)** | **Usual-care**  **Group**  **(n=157)** | ***p*-value ^a^** |
| --- | --- | --- | --- |
| **Maternal outcomes** |  |  |  |
| Gestational hypertension (n=260)*, no. (%) | 1 (0.8) | 5 (3.7) | 0.115 |
| Emergency Cesarean section rate, no. (%) | 45 (29.4) | 45 (28.7) | 0.884 |
| Weight gain at delivery (kg), median (IQR) | 11(7, 15) | 10 (7, 15) | 0.950 |
| Body weight at delivery (kg), median (IQR) | 86.8 ±16.5 | 86.9 ±14.7 | 0.950 |
| Maternal admission day (day), median (IQR) | 3 (3, 4) | 3 (3, 4) | 1.000 |
| **Fetal outcomes** |  |  |  |
| Fetal growth restriction ^a^, no. (%) | 3 (2.0) | 3 (1.9) | 0.975 |
| Gestational age at delivery (week), mean (SD) | 38.0±2.1 | 37.8±2.3 | 0.497 |
| Preterm birth ^b^, no. (%) | 12 (7.8) | 16 (10.2) | 0.471 |
| Infant birthweight (gram), mean (SD) | 3137.6 ±576.2 | 3097.6± 593.9 | 0.548 |
| Low infant birthweight (Birthweight <2500  gram) ^c^, no. (%) | 16(10.5) | 15(9.6) | 0.791 |
| Large for gestational age (birthweight≥4000  gram) ^d^, no. (%) | 4(2.6) | 5(3.2) | 0.765 |
| Small for gestational age ^e^, no. (%) | 6 (3.9) | 4 (2.6) | 0.494 |
| Fetal respiratory distress, no. (%) | 9 (8.9) | 9 (9.1) | 0.96 |
| APGAR score, 1 minute, mean (SD) | 8.3±1.2 | 8.1±1.3 | 0.319 |
| APGAR score, 5 minutes, mean (SD) | 9.3±1.1 | 9.3±0.7 | 0.937 |
| Neonatal admission day (day), median (IQR) | 3 (3, 4) | 3 (3, 4) | 1.0 |
| Neonatal intensive care admission, no. (%) | 17(11.1) | 18 (11.5) | 0.922 |
| Composite outcome 1 ^f^, no. (%) | 21 (13.7) | 39 (24.8) | 0.012 |
| Composite outcome 2 ^g^, no. (%) | 58 (37.9) | 70 (44.6) | 0.231 |
| Composite outcome 3 ^h^, no. (%) | 24 (15.7) | 36 (22.9) | 0.104 |

**Abbreviations:** CPAP, continuous positive airway pressure; IQR, interquartile range; SD, standard deviation

* Analysis excluding 50 participants with preexisting chronic hypertension

^a^ Fetal growth restriction was defined as a weight <10^th^ centile for gestational age during intrauterine period.

^b^ Preterm birth was defined as [birth](https://en.wikipedia.org/wiki/Birth) of a newborn at less than 37 weeks [gestational age](https://en.wikipedia.org/wiki/Gestational_age).

^c^ Low infant birthweight was defined as infant birthweight <2500 gram regardless of gestational age.

^d^ Large for gestational age was defined as infant birthweight ≥4000 gram.

^e^ Small for gestational age was defined as infant birthweight < 10 centile for gestational age

^f^ The composite 1 endpoint included hypertensive disorders in pregnancy comprised of preeclampsia or gestational hypertension

^g^ The composite 2 endpoint included hypertensive disorder of pregnancy comprised of preeclampsia and gestational hypertension, preterm birth, fetal growth restriction, or emergency cesarean section.

^h^ The composite 3 endpoint included preterm birth, fetal growth restriction, severe preeclampsia, eclampsia, or death.

| **Events ^a^, no. (%)** | **CPAP Group**  N=153 | **Usual-care group**  N=157 | ***p*-value** |
| --- | --- | --- | --- |
| **Maternal events, no. (%)** |  |  |  |
| Upper respiratory tract infection | 1 (0.7) | 0 (0) | 0.494 |
| Lower respiratory tract infection | 0 (0) | 0 (0) |  |
| Asthma | 6 (3.9) | 6 (3.8) | 1.0 |
| Allergic rhinitis | 27 (17.7) | 22 (14.0) | 0.381 |
| Urinary tract infection | 1 (0.7) | 0 (0) | 0.494 |
| Gastrointestinal tract infection | 1 (0.7) | 1 (0.6) | 1.0 |
| Wound infections | 1 (0.7) | 1 (0.6) | 1.0 |
| **Obstetric complication, no. (%)** |  |  |  |
| Placenta previa | 0 (0) | 1 (0.6) | 1.0 |
| Polyhydramnios | 1 (0.7) | 0 (0) | 0.494 |
| Breech presentation | 0 (0) | 4 (2.6) | 0.123 |
| Cephalo-pelvic disproportion | 5 (3.3) | 4 (2.6) | 0.748 |
| Unfavorable cervix | 0 (0) | 2 (1.3) | 0.498 |
| Failed induction | 0 (0) | 1(0.6) | 1.0 |
| Premature rupture of membrane | 2 (1.3) | 2 (1.3) | 1.0 |
| Retained placenta | 1(0.7) | 1 (0.6) | 1.0 |
| Post-partum hemorrhage | 2 (1.3) | 3 (1.9) | 1.0 |
| Wound infections | 1 (0.7) | 1 (0.6) | 0.494 |
| **Fetal complication, no. (%)** |  |  |  |
| Meconium aspiration syndrome | 1 (0.7) | 1 (0.6) | 1.0 |
| Neonatal jaundice | 11 (7.2) | 13 (8.3) | 0.719 |
| Neonatal sepsis | 5 (3.3) | 1 (0.6) | 0.117 |
| Neonatal hypoglycemia | 12 (7.8) | 8 (5.1) | 0.362 |
| Polycythemia | 1 (0.7) | 1 (0.6) | 1.0 |

**Table S5. Adverse events by intervention groups**

**Abbreviations:** CPAP, continuous positive airway pressure

**^a^** The chi-square test was used to compare the difference in the proportions of participants experiencing adverse outcomes between the CPAP versus usual-care group.

**Table S6. Primary outcomes on blood pressures using the per-protocol and counterfactual analyses**

| ***Per-protocol Analysis*** | | | | | | | | | | | | |
| --- | --- | --- | --- | --- | --- | --- | --- | --- | --- | --- | --- | --- |
|  | **CPAP (n=50)** | | | **Usual-care (n=155)** | | | **Overall mean** | | **Mean**  **difference**  **(95%CI) ^c^** | ***p-*value** | **Adjusted**  **Mean**  **Difference**  **(95%CI) ^d^** | ***p-*value** |
| **Mean**  **(SE)^a^**  **mmHg** | **18-20**  **weeks** ^b^ | **24-28**  **weeks** | **32-34**  **weeks** | **18-20**  **weeks** ^b^ | **24-28**  **Weeks** | **32-34**  **weeks** | **CPAP** **vs** **Control** | |  |  |  |  |
| **SBP** | 112.7(1.7) | 110.3(1.7 | 113.6(1.7) | 116.5(1.0) | 114.1(1.0) | 117.4(1.0) | 112.2 (1.6) | 116.0 (0.9) | -3.8  (-7.4, -0.1) | 0.043 | -4.25  (-7.46, -1.03) | 0.010 |
| **DBP** | 69.4(1.1) | 67.8(1.1) | 70.6(1.1) | 72.7(0.7) | 71.0(0.7) | 73.8(0.7) | 69.3 (1.1) | 72.5 (0.6) | -3.2  (-5.7, -0.8) | 0.009 | -3.64  (-5.67, -1.60) | <0.001 |
| **MAP** | 83.9 (1.2) | 82.0(1.2) | 84.9 (1.3) | 87.3(0.8) | 85.4 (0.8) | 88.3 (0.8) | 83.6 (1.2) | 87.0 (0.7) | -3.4  (-6.1, -0.7) | 0.013 | -3.85  (-6.14, -1.55) | 0.001 |
| ***Counterfactual Analysis*** | | | | | | | | | | | | |
| **Mean**  **(SE) ^a^**  **mmHg** | **CPAP (n=52)** | | | **Usual-care (n=258)** | | | **Overall mean** | | **Mean**  **difference**  **(95%CI) ^c^** | ***p-*value** | **Adjusted**  **Mean**  **Difference**  **(95%CI) ^d^** | ***p-*value** |
|  | **18-20**  **weeks** ^b^ | **24-28**  **weeks** | **32-34**  **weeks** | **18-20**  **weeks** ^b^ | **24-28**  **Weeks** | **32-34**  **weeks** | **CPAP** **vs** **Control** | |  |  |  |  |
| **SBP** | 111.0(3.5) | 108.7(3.5) | 111.5(3.5) | 116.7(1.0) | 114.4(1.0) | 117.2(1.0) | 110.4 (3.5) | 116.1 (1.0) | -5.7  (13.8, 2.4) | 0.169 | -6.35  (-13.68, 0.97) | 0.089 |
| **DBP** | 66.2(2.4) | 65.1(2.4) | 67.1(2.4) | 72.6(0.7) | 71.5(0.7) | 73.5(0.7) | 66.2 (2.4) | 72.5 (0.6) | -6.4  (-11.8, -0.9) | 0.022 | -6.76  (-11.50, -2.01) | 0.005 |
| **MAP** | 81.1(2.6) | 79.7(2.6) | 81.9(2.6) | 87.3(0.8) | 85.8(0.8) | 88.1(0.8) | 80.9 (2.6) | 87.1 (0.7) | -6.2  (-12.2, -0.1) | 0.046 | -6.63  (-11.91, -1.35) | 0.014 |

**Abbreviations:** CPAP, continuous positive airway pressure; DBP, diastolic blood pressure; MAP, mean arterial pressure; SBP, systolic blood pressure SE, standard error; 95%CI, 95% confidence interval

^a^ Primary outcome on systolic and diastolic blood pressures are shown as means and SE.

^b^ Blood pressure data during 18-20 weeks gestation from participants who were randomized during 2^nd^ trimester in both CPAP (n=6) and usual-care groups (n=6) were not included in the analyses

^c^ Intergroup difference is calculated as change in CPAP group compared to that usual-care group using longitudinal data analysis with mixed-effect model after randomization presented as mean difference and 95% CI.

^d^ Adjusted intergroup difference is calculated as difference of marginal mean between CPAP compared to the usual-care groups using a mixed-effect linear regression model after randomization presented as mean difference and 95%CI adjusted with underlying chronic hypertension status, anti-hypertensive medication use and gestational age.

**Table S7. Post-hoc analyses according to participants with mild OSA/UARS and OSA subgroups on primary outcomes of blood pressures using modified intention-to-treat analysis**

| **Modified-intention-to-treat analysis ^*^** | | | | | | | | |
| --- | --- | --- | --- | --- | --- | --- | --- | --- |
|  | **Mild OSA/UARS (n=93) ^a^**  **CPAP (n=43) vs Usual-care (n=50)** | | | | **OSA (n=217) ^a^**  **CPAP (n=110) vs Usual-care (n=107)** | | | |
| **Blood pressure (mmHg)** | **Crude difference^b^** | ***p*-value** | **Adjusted difference^c^** | ***p*-value** | **Crude difference^b^** | **p-value** | **Adjusted difference^c^** | ***p-*value** |
| **Systolic blood pressure** | -3.28 | 0.171 | -2.92 | 0.16 | -1.71 | 0.271 | -1.78 | 0.187 |
| **Diastolic blood pressure** | -2.93 | 0.06 | -2.63 | 0.04 | -2.05 | 0.046 | -2.11 | 0.013 |
| **Mean blood pressure** | -3.05 | 0.08 | -2.73 | 0.06 | -1.93 | 0.091 | -2.00 | 0.035 |

**Abbreviations:** AHI, apnea-hypopnea index; CPAP, continuous positive airway pressure; OSA, obstructive sleep apnea; RDI, respiratory disturbance index; UARS, upper airway resistance syndrome

^a^ All participants had respiratory disturbance index (RDI)≥5 events/hour based on the inclusion criteria regardless of AHI. AHI<5 and RDI≥5 is defined as Mild OSA/UARS; AHI≥5 is defined as OSA.

^b^ Intergroup crude difference is calculated as change in CPAP group compared to that usual-care group using longitudinal data analysis with mixed-effect model after randomization presented as mean difference.

^c^ Intergroup adjusted difference is calculated as change in CPAP group compared to that usual-care group using longitudinal data analysis with mixed-effect model after randomization presented as mean difference and adjusted with underlying hypertension status, anti-hypertensive medication use, and obesity.

**^*^ Modified intention-to-treat analysis**:

Mild OSA/UARS subgroup (N=93) comprised of 50 usual-care and 43 CPAP participants with 14 (32.6%) CPAP-adherence.

OSA subgroup (N=217) comprised of 107 usual-care and 110 CPAP participants with 36 (32.7%) CPAP-adherence.

**Table S8. Post-hoc analyses according to participants with mild OSA/UARS and OSA subgroups on secondary outcomes of preeclampsia and pregnancy-induced hypertension using modified intention-to treat analysis**

| **Modified Intention to treat analysis** | **CPAP**  **n (%)** | **Usual-care Group**  **n (%)** | **Risk difference ^b^ % (95%CI)** | **Number needed to treat^b^**  **(95%CI)** | ***p*-value** | **Adjusted risk difference ^c^ % (95%CI)** | **Adjusted number needed to treat ^c^ (95%CI)** | ***p*-value** |
| --- | --- | --- | --- | --- | --- | --- | --- | --- |
| **Preeclampsia** |  |  |  |  |  |  |  |  |
| Mild OSA/UARS (n=93) ^a^ | 4/43 (9.30) | 8/50 (16.0) | -7%  (-20%, 7%) | 15  (-14, 55) | 0.326 | -13%  (-25%, -0.4%) | 8  (1, 15) | 0.044 |
| OSA (n=217) | 16/110 (14.55) | 27/107 (25.53) | -11%  (-21%, -1%) | 9  (1, 19) | 0.047 | -9%  (-11%, -7%) | 11  (9, 14) | <0.001 |
| **Hypertensive disorders in pregnancy** |  |  |  |  |  |  |  |  |
| Mild OSA/UARS (n=93) ^a^ | 4/43 (9.30) | 8/50 (16.0) | -7%  (-20%, 7%) | 15  (-15, 45) | 0.326 | -13%  (-25%, -0.4%) | 8  (1. 15) | 0.044 |
| OSA (n=217) | 17/110 (15.45) | 31/107 (28.97) | -14%  (-24%, -3%) | 7  (1, 13) | 0.015 | -11%  (-13%, -9%) | 9  (7, 11) | <0.001 |

**Abbreviations:** AHI, apnea-hypopnea index; CPAP, continuous positive airway pressure; OSA, obstructive sleep apnea RDI, respiratory disturbance index; UARS, upper airway resistance syndrome

^a^ All participants had respiratory disturbance index (RDI)≥5 events/hour based on the inclusion criteria regardless of AHI. AHI<5 and RDI≥5 is defined as Mild OSA/UARS; AHI≥5 is defined as OSA. ^b^ Binary logistic regression analysis was used to calculate the risk difference and number needed to treat of preeclampsia and hypertensive disorders in pregnancy between participants in CPAP versus usual-care groups. ^c^ Multivariate logistic regression analysis adjusted with covariates (underlying hypertension and diabetes, nulliparous status and site of study) was used to calculated the adjusted risk difference and adjusted number needed to treat of pregnancy outcomes between participants in CPAP versus usual-care groups.

**Table S9. A sensitivity analysis excluding 12 participants with new-onset OSA randomized during 2^nd^ trimester on primary outcomes of blood pressures using modified intention-to treat analysis**

| **Modified intention-to-treat analysis** | | | | | | | | | | |
| --- | --- | --- | --- | --- | --- | --- | --- | --- | --- | --- |
| **Mean**  **(SE)^a^**  **mmHg** | **CPAP (n=147)** | | | | **Usual-care Group (n=151)** | | | | **Intergroup**  **Difference**  **(95%CI)** ^c^ | ***p*-value** |
|  | **Baseline** | **18-20**  **weeks** ^b^ | **24-28**  **weeks** | **32-34**  **Weeks** | **Baseline** | **18-20**  **weeks** ^b^ | **24-28**  **weeks** | **32-34**  **weeks** |  |  |
| **SBP** | 117.1(1.3) | 114.7(1.0) | 112.5(1.0) | 115.2(1.0) | 116.2 (1.08) | 116.7(1.0) | 114.4(1.0) | 117.2(1.0) | -1.8  (-4.5, 0.9) | 0.190 |
| **DBP** | 73.7(0.94) | 70.4(0.7) | 69.3(0.7) | 71.3(0.7) | 74.0(0.76) | 72.6(0.7) | 71.5(0.7) | 73.8(0.7) | -2.0  (-3.8, -0.2) | 0.027 |
| **MAP** | 88.2(1.03) | 85.2 (0.8) | 83.7 (0.8) | 86.0 (0.8) | 88.0(0.83) | 87.3(0.7) | 85.8(0.7) | 88.0(0.7) | -1.9  (-3.9, -0.1) | 0.057 |

**Abbreviations:** CPAP, continuous positive airway pressure; DBP, diastolic blood pressure; MAP, mean arterial pressure; SBP, systolic blood pressure; SE, standard error; 95%CI, 95% confidence interval

^a^ Primary outcome on systolic and diastolic blood pressures are shown as means and SE estimated at each time point from the mixed-effect linear regression.

^b^ Blood pressure data during 18-20 weeks gestation from participants who were randomized during 2^nd^ trimester in both CPAP (n=6) and usual-care groups (n=6) were not included in the analyses

^c^ Intergroup difference is calculated as difference of marginal means (overall mean) between CPAP compared to the usual-care groups using mixed-effect linear regression model after randomization presented as mean difference and 95% CI.

**Table S10. A sensitivity analysis excluding participants with new-onset OSA randomized during 2^nd^ trimester on secondary outcomes of preeclampsia and hypertensive disorders in pregnancy using modified intention-to treat analysis**

| **Endpoints** | **CPAP Group** | **Usual-care Group** | **Risk difference**  **% ^a^**  **(95%CI)** | **Number needed to treat ^a^**  **(95%CI)** | ***p*-value** |
| --- | --- | --- | --- | --- | --- |
| ***Modified intention-to-treat analysis*** | ***(n=147)*** | ***(n=151)*** |  |  |  |
| **Preeclampsia, no. (%)** | 20 (13.61) | 35 (23.18) | -10  (-18, -1) | 10  (1, 20) | 0.031 |
| **Severe preeclampsia ^b^** | 13 (8.84) | 22 (13.91) | -5  (-12, 21) | - | 0.167 |
| **Early-onset preeclampsia ^c^** | 4 (2.72) | 4 (2.65) | 0.1  (-4, 3.5) | - | 0.969 |
| **Late-onset preeclampsia ^d^** | 16 (10.88) | 31 (20.53) | -10  (-18, -1.5) | 10  (2, 19) | 0.021 |
| **Hypertensive disorders in pregnancy,**  **no. (%)^e^** | 21 (14.29) | 39 (25.83) | -12  (-21, -3) | 9  (2, 15) | 0.015 |

**Abbreviations:** CPAP, continuous positive airway pressure; 95%CI, 95% confidence interval

^a^ Binary logistic regression analysis was used to calculate the risk difference and number needed to treat of preeclampsia and hypertensive disorders in pregnancy between participants in CPAP versus usual-care groups.

^b^ Severe preeclampsia was defined according to Report of the American College of Obstetricians and Gynecologists’ Task Force on hypertension in pregnancy^31-32^.

^c^ Early-onset preeclampsia was defined as developing preeclampsia before 34 completed weeks’ gestation;

^d^ Late-onset preeclampsia was defined as developing preeclampsia a or beyond 34 weeks’ gestation.

^e^  Hypertensive disorders in pregnancy comprised of preeclampsia and gestational hypertension
